# The AGO2 adaptor LIMD1 expands the functional and evolutionary reach of microRNA targeting

**DOI:** 10.1126/sciadv.aed6875

**Published:** 2026-07-23

**Authors:** Alex F. F. Crozier, Kunal M. Shah, Paul Grevitt, Anisha Thind, Eleni Maniati, Jun Wang, Kylie Shen, Diana Cox, Vinothini Rajeeve, Pedro Cutillas, Dimitris Lagos, Faraz Mardakheh, Sam Griffiths-Jones, Antonio Marco, Tyson V. Sharp

**Affiliations:** ^1^ Centre for Cancer Cell and Molecular Biology, Barts Cancer Institute, Queen Mary University of London, London, UK.; ^2^ Centre for Cancer Biomarkers and Biotherapeutics, Barts Cancer Institute, Queen Mary University of London, London, UK.; ^3^ Eclipse BioInnovations, San Diego, CA, USA.; ^4^ Cell Signalling and Proteomics Laboratory, Centre for Cancer Evolution, Barts Cancer Institute, Queen Mary University of London, London, UK.; ^5^ York Biomedical Research Institute, University of York, York, UK.; ^6^ Department of Biochemistry, University of Oxford, South Parks Road, Oxford, UK.; ^7^ School of Biological Sciences, Faculty of Biology, Medicine and Health, University of Manchester, Manchester, UK.; ^8^ School of Life Sciences, University of Essex, Colchester, UK.

## Abstract

MicroRNA (miRNA) silencing is classically ascribed to RNA-sequence rules that guide Argonaute 2 (AGO2) targeting. Using chimeric eCLIP and complementary analyses in CRISPR-edited human lung epithelial cells, we show that efficient miRNA targeting also depends on the AGO2 adaptor-scaffold LIMD1. In LIMD1-deficient cells, AGO2 binds more miRNAs, but each AGO2-miRNA engages fewer transcripts and sites, reducing occupancy and halving both the breadth and depth of targeting. LIMD1 dependence is most pronounced for poorly conserved, weakly seed-paired sites that nonetheless form stable duplexes. LIMD1 deficiency alters AGO2 footprints and derepresses oncogenic targets inversely correlated with LIMD1 expression in lung adenocarcinoma. Thus, LIMD1 modifies the outcome of sequence-defined interactions that would otherwise be infrequent, unstable, or unproductive, revealing an adaptor-governed layer of posttranscriptional regulation beyond RNA-sequence rules.

## Teaser

LIMD1 helps AGO2 silence microRNA targets more effectively, revealing an additional layer of control beyond RNA sequence alone.

## INTRODUCTION

MicroRNAs (miRNAs) are small noncoding RNAs that repress gene expression posttranscriptionally by guiding Argonaute (AGO) proteins to target sites in mRNAs (*1*). Each miRNA can regulate many transcripts, and most human mRNAs carry conserved sites, creating dense regulatory networks that shape development, homeostasis, and disease (*2*). Canonical targeting is dictated primarily by seed complementarity, modulated by flanking context, supplementary pairing, and structure; noncanonical sites further broaden targeting (*1*, *3*–*8*). AGO-miRNA:target complexes recruit trinucleotide repeat–containing 6 (TNRC6) scaffolds and associated cofactors to form the miRNA-induced silencing complex (miRISC), coupling recognition to translational repression and mRNA decay (*9*–*11*). Yet despite extensive study, the rules governing targeting efficacy remain incomplete, limiting mechanistic insight and therapeutic progress (*12*).

Although base pairing explains site recognition, repression efficacy also reflects miRISC architecture (*13*, *14*). AGO-miRNAs are dynamic, undergoing conformational remodeling upon target binding and release; posttranslational modifications, cooperativity via TNRC6 multivalency, and paralog choice modulate dwell time, specificity, and efficacy (*14*–*24*). These features support a model in which RNA base-pairing rules integrate with dynamic miRISC architecture to define repression specificity, strength, and kinetics (*13*, *15*, *25*, *26*). Yet most studies of targeting remain RNA centric (*12*); despite recent advances, predictors of binding and repression still only account for around half of the variability in miRNA-mediated repression (*8*), implying that there are additional, undefined determinants of targeting effectiveness (*27*). Likewise, evolutionary analyses continue to emphasize miRNA and site conservation (*2*, *28*, *29*), leaving the roles of miRISC adaptor proteins in targeting and regulatory evolution underexplored.

**Fig. 1.**
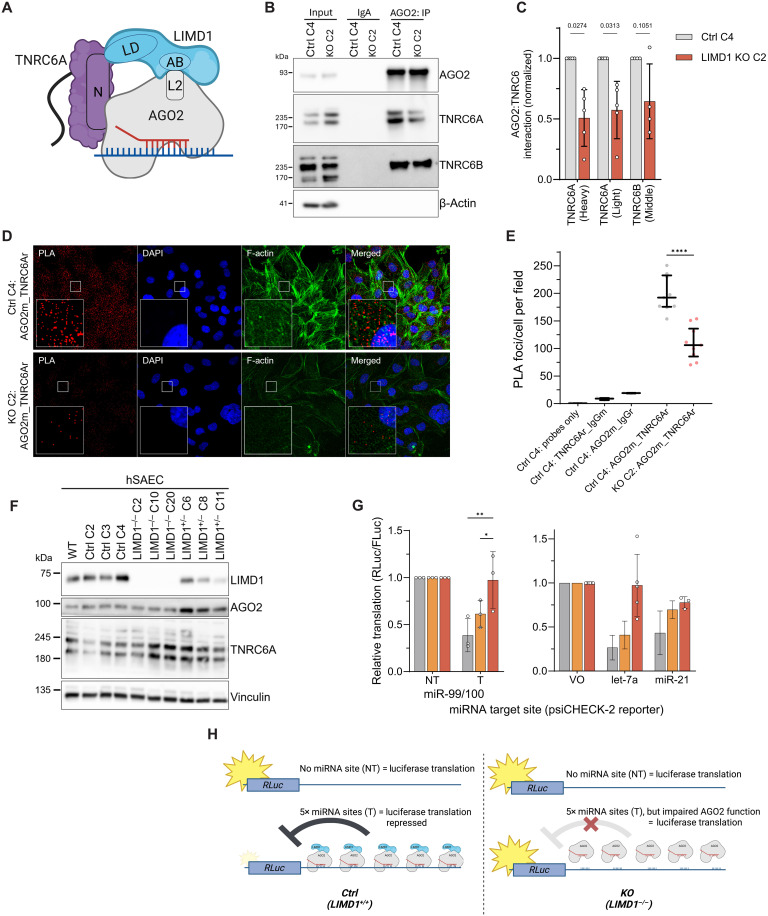
LIMD1 potentiates AGO2-TNRC6A interactions and miRNA-reporter repression in hSAECs. (**A**) Schematic summarizing experimentally defined LIMD1-AGO2-TNRC6A interaction interfaces from Bridge *et al.* (*19*). LIMD1 binds AGO2 via a pre-LIM AGO-binding (AB) motif (amino acids 140 to 166) and TNRC6A via its LIM domains (LD), thereby promoting productive miRISC assembly. Functional rescue assays showed that WT LIMD1 and Δ186-260 restored silencing, whereas deletion of the AB motif (Δ140-166) abolished AGO2 interaction and failed to rescue. L2, AGO2 linker-2 domain; AB, AGO-binding motif; LD, LIM domains; N, TNRC6A N-terminal region. (**B** and **C**) Co-IP showing reduced AGO2-TNRC6A association in KO C2 relative to Ctrl C4 cells; the difference in AGO2-TNRC6B association did not reach statistical significance (means ± SD, *n* ≥ 4; unpaired *t* test with correction). (**D** and **E**) In situ proximity ligation assay (PLA) of AGO2:TNRC6A showing reduced proximal association (<∼40 nm) in LIMD1 KO C2 versus Ctrl C4 cells. (D) Representative fluorescence images showing PLA puncta (red), nuclei [4′,6-diamidino-2-phenylindole (DAPI), blue], and F-actin (green); negative controls are shown in fig. S1A. Insets show 10× boxed regions. The PLA channel was thresholded for clarity. (E) Quantification of PLA foci per cell averaged per imaging field (*n* ≥ 3). Each dot represents one field. Welch’s two-tailed *t* test on field means; ****P < 0.0001. (**F**) CRISPR-Cas9 editing of hSAECs generated Ctrl (LIMD1^+/+^), Het (LIMD1^+/−^), and KO (LIMD1^−/−^) clones (*n* = 3 per genotype). (**G** and **H**) Dual-luciferase assays showing reduced repression of psiCHECK-2 reporters bearing synthetic miRNA sites (miR-99/100, let-7a, miR-21), with a dose-dependent trend from Ctrl_C4 to Het_C6 to KO_C2. Nontargeting (NT) or vector-only (VO) reporters served as controls. Data are means ± SD from ≥3 experiments; two-way analysis of variance (ANOVA) with correction. (H) Schematic of the psiCHECK-2 reporter system showing targeting (T) and NT miR-99/100 tandem reporters containing five repeats.

LIM domain containing 1 (LIMD1), a metazoan adaptor-scaffold with tumor-suppressive activity in lung cancer (*30*–*32*), provides a rare example of a miRISC-associated adaptor with experimentally defined molecular interaction interfaces (*19*, *33*). Prior work showed that LIMD1 associates endogenously with AGO2 and TNRC6A to promote formation of silencing-competent miRISCs: LIMD1 loss reduces but does not abolish AGO2-TNRC6A association and impairs miRNA reporter silencing (*19*, *33*). Domain mapping and direct binding assays identified a short AGO-binding motif in the LIMD1 pre-LIM region (amino acids 140 to 166) that is necessary and sufficient for engagement of the AGO2 linker-2 (L2) domain; notably, the isolated 140-166 fragment was shown to bind purified AGO2 in vitro. The C-terminal LIM domains bind the TNRC6A N terminus, thereby bridging AGO2 and TNRC6A through separable interfaces (Fig. 1A) (*19*). Consistent with a direct architectural role, rescue experiments demonstrated that deletion of the AGO-binding motif (Δ140-166) abolished AGO2 interaction and failed to restore silencing, whereas control deletions that retained AGO2 binding remained competent (*19*). Together, these findings established LIMD1 as a miRISC-associated AGO2 adaptor that promotes formation of silencing-competent complexes and miRNA-mediated reporter silencing in cells. Whether this adaptor dependence generalizes across endogenous AGO2-miRNA targeting networks and which specific miRNA:mRNA interaction features may confer LIMD1 dependence have remained unknown.

To address this, we combine AGO2 chimeric enhanced cross-linking and immunoprecipitation (eCLIP) (*34*) with transcriptomic, proteomic, motif, evolutionary, and thermodynamic analyses in primary human small airway epithelial cells (hSAECs) across graded *LIMD1* expression states. By interrogating AGO2-miRNA:target interactions across scales ranging from global targeting patterns to individual miRNA:mRNA duplex features, we test whether perturbation of a defined AGO2 adaptor alters targeting breadth, occupancy, and regulatory output, and whether specific RNA interaction features predict adaptor dependence. Our findings demonstrate that LIMD1 potentiates efficient AGO2-miRNA engagement with endogenous targets and modulates repression magnitude transcriptome-wide. This supports a model whereby adaptor incorporation into miRISC contributes to targeting behavior alongside sequence-encoded RNA pairing rules, with implications for how posttranscriptional regulation is tuned in physiology and disease.

## RESULTS

### LIMD1 potentiates AGO2-TNRC6A interactions and miRNA-reporter repression

We first sought to corroborate our previous findings from HeLa cells in a cell model with greater physiological and disease relevance. Reduced *LIMD1* expression, frequently via clonal loss of heterozygosity (LOH), is pervasive in non–small cell lung cancer (NSCLC) and predicts poor survival (*30*, *31*, *35*). The miRISC assembly state and dynamics in normal tissue are also known to be distinct from those in transformed cancer cell lines (*14*, *36*–*38*). We therefore used immortalized, nontransformed hSAECs, a diploid alveolar type II–derived primary line providing a lineage-matched model of lung adenocarcinoma (LUAD) (*39*, *40*), to study LIMD1’s role in miRNA silencing in a more physiological and disease-relevant context.

Consistent with our previous work in HeLa cells (*19*), coimmunoprecipitation (Co-IP) and in situ proximity ligation assays (PLAs) performed in representative control (Ctrl) and KO hSAEC clones showed that complete LIMD1 loss reduces AGO2-TNRC6A interaction; Co-IP analysis of AGO2-TNRC6B association did not show a difference that reached statistical significance between Ctrl and KO cells (Fig. 1, A to E, and fig. S1A). We therefore next asked how LIMD1 loss affects miRNA silencing more broadly in this cellular context. To this end, we used CRISPR-Cas9 gene editing to generate three independent hSAEC clones per genotype: *LIMD1* knockout [KO; (C2, C10, and C20)], heterozygous [Het; (C6, C8, and C11), modeling LOH], and guide Ctrl (C2, C3, and C4) cells (Fig. 1F and fig. S1B). Genome sequencing confirmed on-target editing with minimal detectable off-target effects (tables S1 to S3). Unsupervised clustering of transcriptomes showed that CRISPR clones grouped by genotype, supporting the reproducibility of genotype-associated transcriptional changes (fig. S1C). AGO2 protein abundance was unchanged in KO and increased in Het, whereas TNRC6A levels were elevated in both (fig. S1D), excluding reduced AGO2 or TNRC6A protein abundance as an explanation for impaired silencing. Consistent with reduced AGO2-TNRC6A interaction, miRNA reporters performed in Ctrl C4, Het C6, and KO C2 cells showed dose-dependent target derepression (Fig. 1, G and H).

Together, these findings confirm that LIMD1 promotes AGO2-TNRC6A interaction in a diploid, nontransformed airway model and demonstrate a dose-dependent impairment of miRNA reporter silencing upon LIMD1 loss. Building on this reproduced defect in miRISC assembly, and the previous LIMD1-AGO2 interaction framework, we next used multiomics to comprehensively examine how LIMD1 deficiency affects endogenous AGO2-RNA interactions across the transcriptome.

### LIMD1 deficiency increases AGO2-miRNA interactions

We profiled AGO2-bound RNAs across the hSAEC panel using AGO2 chimeric eCLIP (fig. S2A and tables S4 and S5) (*34*). For conventional eCLIP analyses of AGO2-miRNA and AGO2-mRNA interactions, AGO2 clusters were identified in immunoprecipitation (IP) samples using CLIPper, normalized to paired input controls, and classified as AGO2 peaks based on enrichment criteria (log_2_ fold enrichment ≥ 3; *P* ≤ 0.001). Reproducible peaks were defined by irreproducible discovery rate (IDR) analysis across biological replicates (*41*). All subsequent analyses were restricted to these peaks within each genotype, thereby minimizing clone- and replicate-specific effects.

**Fig. 2.**
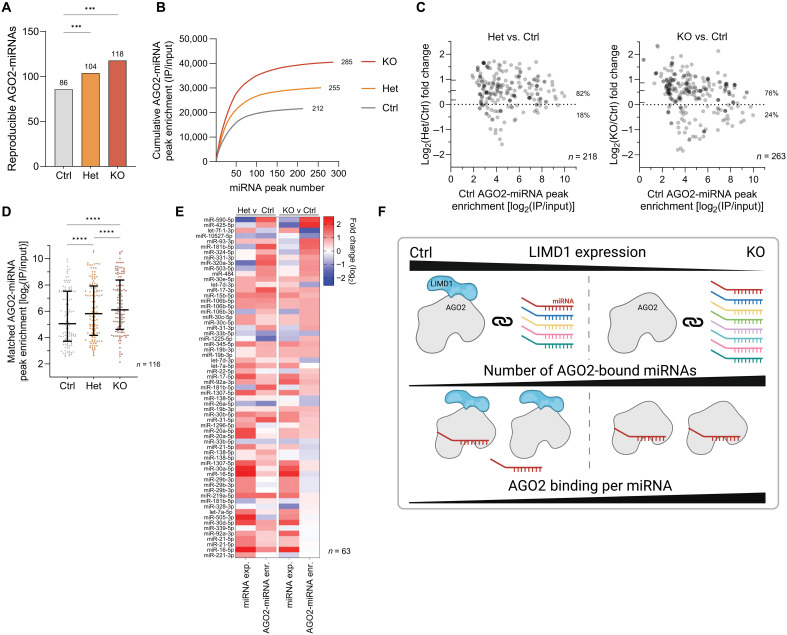
LIMD1 deficiency increases AGO2-miRNA interactions. AGO2-eCLIP analysis of reproducible miRNA peaks in hSAECs across independent CRISPR clones. (**A**) AGO2-miRNA repertoire expands with LIMD1 deficiency (pairwise proportion test with Benjamini-Hochberg correction; ***P < 0.001). (**B**) Cumulative enrichment is higher in Het and KO cells, indicating dose-dependent increases in AGO2 loading. (**C**) Matched peak enrichment in Het and KO versus Ctrl. Each point represents a peak (*x* axis, mean Ctrl enrichment; *y* axis, fold change from DESeq2). Most peaks are enriched (percentages indicated); ticks to the right of the *y* axis denote median and interquartile range. (**D**) Enrichment for 116 peaks shared by Ctrl, Het, and KO, demonstrating a global, dose-dependent increase in AGO2-miRNA binding (Wilcoxon matched-pairs signed-rank test with multiple-comparison correction; ****P < 0.0001). (**E**) Heatmap of log_2_ fold changes in mature miRNA expression and AGO2 peak enrichment per miRNA, ranked by KO versus Ctrl AGO2-miRNA enrichment *P*-adjusted value, showing overall increases in both without clear relation between the two. (**F**) Schematic illustrating LIMD1 dose-dependent increases in the AGO2-miRNA repertoire and binding per miRNA in Het and KO cells versus Ctrl.

Focusing first on IDR-defined reproducible AGO2-miRNA peaks derived from conventional eCLIP libraries, AGO2-miRNA binding increased across the *LIMD1* allelic series. Relative to Ctrl, LIMD1-deficient cells (Het and/or KO) exhibited an expanded set of AGO2-bound miRNAs (Ctrl, 86; Het, 104; and KO, 118), higher total AGO2-miRNA enrichment (Het, +39%; and KO, +88%), and greater enrichment per peak and per miRNA (Fig. 2, A to D; and fig. S2, B to F).

These changes were not explained by miRNA abundance. Although global miRNA expression increased in LIMD1-deficient cells, expression responses across individual miRNAs were heterogeneous and did not positively correlate with changes in AGO2 enrichment (Fig. 2E and fig. S2, G to I). Likewise, within each genotype, absolute miRNA expression did not correlate with AGO2 enrichment (fig. S2J), consistent with AGO2-miRNA binding not simply scaling with miRNA abundance. Notably, both miRNA expression changes and AGO2 enrichment changes were strongly correlated between Het and KO relative to Ctrl (fig. S2, K and L), indicating a coherent, LIMD1-dependent shift in miRNA expression and AGO2-miRNA association. Together, these findings show that LIMD1 deficiency increases AGO2-miRNA association independently of miRNA abundance, consistent with altered miRNA loading and/or retention. One possible interpretation is that LIMD1 loss promotes compensatory loading or reduces AGO2 selectivity. In either case, increased AGO2-miRNA association does not necessarily result in productive target engagement.

This increase in AGO2-miRNA association raised a central question for endogenous targeting: Does enhanced AGO2-miRNA binding translate into broader or stronger AGO2 occupancy on mRNA targets, or does LIMD1 loss instead uncouple miRNA loading from productive target binding? We addressed this by analyzing AGO2-mRNA occupancy and, subsequently, chimeric AGO2-miRNA:target interactions at both transcriptome-wide and locus-resolved levels.

### LIMD1 deficiency reduces AGO2-mRNA occupancy

**Fig. 3.**
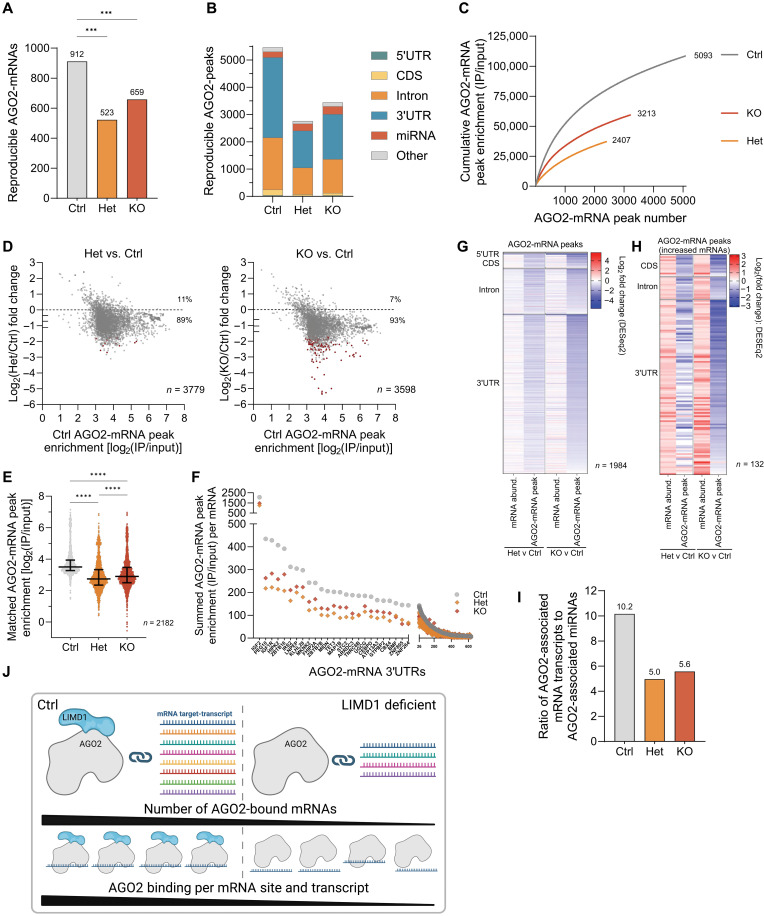
LIMD1 deficiency reduces AGO2-mRNA interactions. AGO2-eCLIP analysis of reproducible mRNA peaks in hSAECs across independent CRISPR clones. (**A**) AGO2-mRNA repertoire contracts with LIMD1 deficiency (pairwise proportion test with Benjamini-Hochberg correction; ***P < 0.001). (**B**) Number and distribution of reproducible peaks by genomic feature (peaks hierarchically annotated as CDS, 5′UTR, 3′UTR, intron, miRNA, and other). (**C**) Reduced total AGO2-mRNA binding in LIMD1-deficient cells indicated by cumulative AGO2-mRNA enrichment. (**D**) Matched peak enrichment in Het and KO versus Ctrl. Each point represents a peak (*x* axis, mean Ctrl enrichment; *y* axis, fold change from DESeq2). Most peaks are de-enriched (percentages indicated); ticks to the right of the *y* axis denote median and interquartile range; significant changes [|log_2_ fold change (log_2_FC)| > 1, *q* < 0.1] are highlighted in red. (**E**) AGO2-mRNA peak enrichment for 2182 peaks common to Ctrl, Het, and KO, showing a global reduction in AGO2-mRNA binding (Wilcoxon matched-pairs signed-rank test with multiple-comparison correction; ****P < 0.0001). (**F**) Summed AGO2-mRNA enrichment across 3′ UTRs per transcript shows reduced binding in Het and KO versus Ctrl. (**G** and **H**) Heatmaps of log_2_FC in mRNA abundance and AGO2-mRNA enrichment (IP/input) for shared peaks, showing widespread loss of AGO2-mRNA binding (G) irrespective of transcript levels and (H) even when mRNAs are increased. Heatmaps are grouped by genomic feature and ordered by loss of binding in KO versus Ctrl. (**I**) Ratio of distinct AGO2-associated mRNAs to miRNAs is halved in LIMD1-deficient cells, indicating uncoupling of miRNA loading from productive mRNA targeting. (**J**) Schematic illustrating reduced AGO2-mRNA repertoire and binding per site and transcript in LIMD1-deficient cells compared with Ctrl.

Despite increased AGO2-miRNA association, analysis of IDR-defined reproducible AGO2-mRNA peaks revealed reduced AGO2 occupancy on mRNAs in LIMD1-deficient cells. Relative to Ctrl, LIMD1-deficient cells exhibited fewer AGO2-associated mRNA transcripts (Ctrl, 912; Het, 523; and KO, 659) and fewer AGO2-mRNA peaks across all mRNA regions, together with reduced total AGO2-mRNA enrichment and lower median enrichment per peak (Fig. 3, A to G; and fig. S3, A to I). DESeq2 analysis showed that per-peak AGO2 enrichment was reduced more strongly in KO than in Het (Het, −39%; and KO, −51%) (Fig. 3, D and G, and fig. S3I). This graded effect is clearer in continuous enrichment measures than in binary peak counts, which depend on thresholded peak detection (*34*, *41*). Reductions were observed transcriptome-wide and across entire mRNAs and mRNA regions (Fig. 3, B, G, and H; and fig. S3, C to I).

Losses in AGO2-mRNA occupancy were not explained by transcript abundance: Global mRNA expression did not decline, correlations between mRNA expression and AGO2 enrichment were weak, and AGO2 enrichment decreased markedly even for transcripts with stable or increased expression (Fig. 3, G and H; and fig. S3, J to L). Thus, independent of RNA abundance, LIMD1 deficiency is associated with reduced AGO2-mRNA occupancy despite greater AGO2-miRNA enrichment, resulting in an approximate halving of the AGO2-mRNA to AGO2-miRNA ratio (Fig. 3, I and J).

### LIMD1 deficiency constrains AGO2-miRNA targeting breadth and depth

We next asked how LIMD1 deficiency alters AGO2-miRNA:target interactions, both transcriptome-wide and at the level of individual miRNAs, targets, and binding events. To address this, we analyzed AGO2 chimeric eCLIP data. Unlike conventional eCLIP, which measures RNA peak enrichment (IP/input), chimeric eCLIP captures ligated AGO2-miRNA:target complexes at nucleotide resolution as chimeric reads, thereby directly identifying endogenous miRNA:target interactions (*34*). Chimeric peaks are genomic clusters of such reads that define discrete AGO2-miRNA:target interaction sites. Reproducible chimeric peaks were restricted to sites supported in each biological replicate within a genotype by ≥3 seed-family–consistent reads, ensuring consistent recovery of specific miRNA:target interactions and minimizing clone- and replicate-level variability; all subsequent chimeric analyses were restricted to these peaks. Individual chimeric reads retain full miRNA-sequence information, allowing interactions to be resolved at the level of individual miRNA species and arms following reproducibility filtering. After unique molecular identifier (UMI) deduplication, each retained read reflects a unique ligation event, corresponding to a single interaction event captured in cells. Thus, each reproducible chimeric peak represents a unique, experimentally defined AGO2-miRNA:target-site interaction with its associated seed match, and the number of chimeric reads within that peak provides a relative measure of interaction frequency within the experiment.

**Fig. 4.**
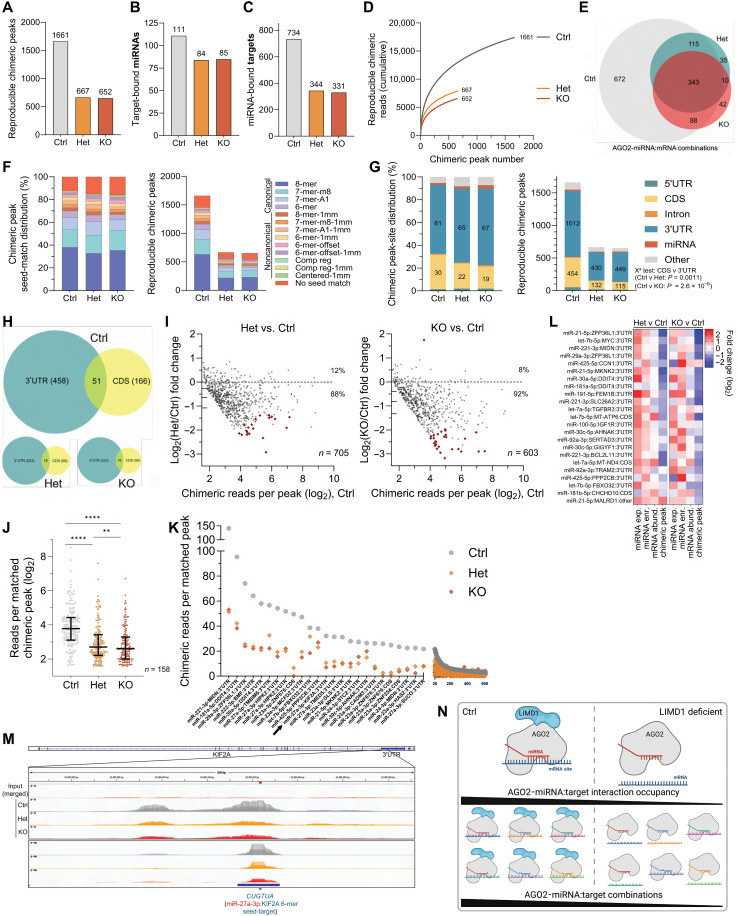
LIMD1 deficiency constrains global AGO2-miRNA targeting. AGO2-eCLIP chimeric analysis in hSAECs across independent CRISPR clones. (**A** to **C**) Numbers of reproducible chimeric peaks (A), target-bound AGO2-miRNAs (B), and AGO2-miRNA–bound transcripts (C) are reduced in LIMD1-deficient cells. (**D**) Cumulative chimeric read counts are lower in Het and KO, indicating fewer AGO2-miRNA:target binding events. (**E**) Venn diagram of unique AGO2-miRNA:mRNA combinations show reduced regulatory breadth. (**F**) Seed-match distribution and chimeric peak numbers are reduced across all pairing classes (TargetScan criteria). (**G**) Genomic feature distribution and number of reproducible chimeric target peaks annotated [chi-square (χ^2^) test for CDS versus 3′UTR peak distribution]. (**H**) Proportional Venn diagrams of AGO2-miRNA targets in 3′UTRs and CDSs indicate distinct regional subnetworks. (**I**) Matched chimeric peaks with ≥3 normalized reads in Ctrl. Each point is a peak (*x* axis, mean Ctrl reads; *y* axis, fold change in Het or KO). Most peaks are de-enriched (percentages indicated); significant changes (|log_2_FC| > 1, *P* < 0.05) are red. (**J**) Normalized chimeric read counts for 158 peaks common to all groups (≥3 reads each), showing global reduction (Wilcoxon matched-pairs signed-rank test with multiple-comparison correction; **P < 0.01; ****P < 0.0001). (**K**) Ranked chimeric reads per peak; the 25 most abundant are labeled by miRNA:mRNA:region. (**L**) Heatmap of log_2_ fold changes in miRNA expression (exp.), AGO2-miRNA enrichment (enr.), mRNA abundance (abund.), and chimeric read counts for peaks with ≥20 Ctrl reads, showing reduced targeting despite increased cognate miRNA expression and AGO2 loading. (**M**) Integrative Genomics Viewer tracks of an 850-bp KIF2A 3′UTR region showing AGO2 IP, input, and chimeric reads by genotype; Het and KO show reduced AGO2 enrichment over input and fewer chimeric reads at the indicated miR-27a-3p:KIF2A 6-mer site. (**N**) Schematic summarizing reduced AGO2-miRNA:target interaction frequency and repertoire in LIMD1-deficient cells.

Analysis of reproducible chimeric peaks across the *LIMD1* allelic series revealed a marked contraction of AGO2-miRNA:target interactions with LIMD1 deficiency. Reproducible chimeric peaks decreased by ∼60% (Ctrl, 1661; Het, 667; and KO, 652), with fewer target-engaged AGO2-miRNAs and a >50% reduction in miRNA-bound transcripts (Fig. 4, A to C). Total chimeric reads and distinct AGO2-miRNA:mRNA combinations also declined by >50% (Fig. 4, D and E, and fig. S4A). Thus, LIMD1 deficiency reduced both the breadth (number of interaction sites and target transcripts) and depth (interaction frequency per site) of AGO2-miRNA targeting.

All seed classes were similarly affected, indicating that reductions were broadly distributed across canonical and noncanonical pairing modes (Fig. 4F and fig. S4B). Losses were observed across transcript regions, with a disproportionately large reduction in coding sequence (CDS) interactions (Fig. 4G and fig. S4, C and D). Minimal overlap between CDS and 3′ untranslated region (3′UTR) chimeric targets suggests distinct, region-specific, LIMD1-sensitive targeting subnetworks (Fig. 4H). Among matched chimeras, median read counts decreased by 36% in Het and 51% in KO (Fig. 4, I and J; and fig. S4, E and F), including for high-frequency interactions and despite increased miRNA expression and AGO2-miRNA enrichment (Fig. 4, K and L; and fig. S4G). An Integrative Genomics Viewer view of the AGO2–miR-27a-3p interaction at the KIF2A 3′UTR 6-mer site, which is not predicted by TargetScan or miRDB, illustrates reduced AGO2 occupancy and fewer chimeric reads in LIMD1-deficient cells (Fig. 4, M and N). This locus-level example mirrors the broader loss of site occupancy and targeting breadth observed genome-wide.

**Fig. 5.**
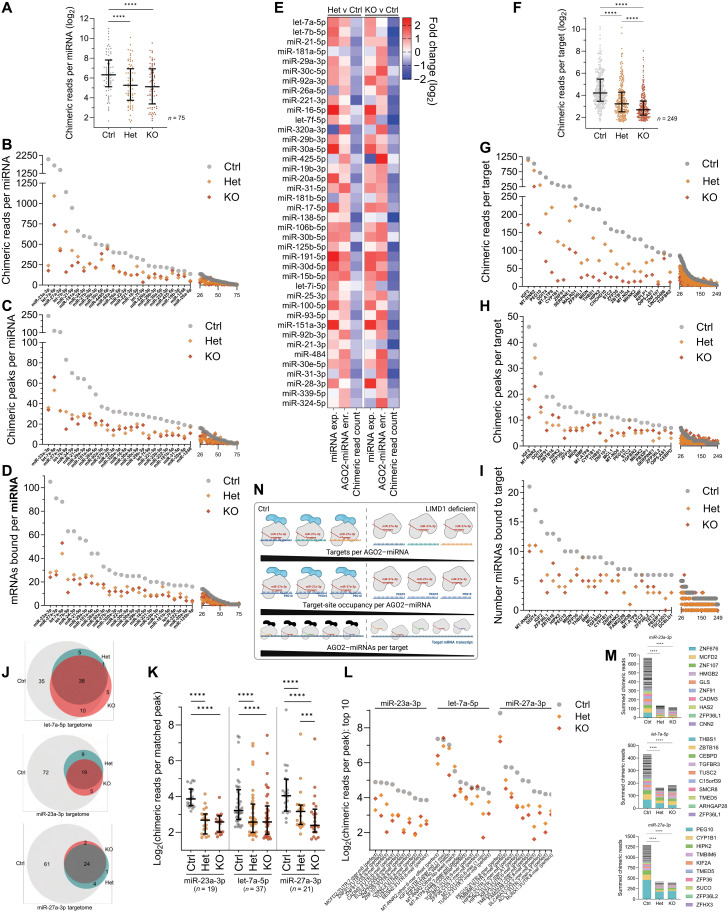
LIMD1 deficiency constrains breadth and depth of targeting by each AGO2-miRNA. Per-miRNA and per-target AGO2-eCLIP-chimeric analysis in hSAECs across independent CRISPR clones. (**A**) Chimeric reads per AGO2-miRNA across reproducibly detected miRNAs show dose-dependent reduction in LIMD1-deficient cells (Wilcoxon matched-pairs signed-rank test; ****P < 0.0001). (**B** to **D**) Expanded views of the top 25 (left) and next 50 (right) AGO2-miRNAs show reduced chimeric reads (B), peaks (C), and target transcripts (D) per miRNA, ordered by counts in Ctrl. (**E**) Fold change (log_2_) in mature miRNA expression, AGO2-miRNA enrichment, and chimeric reads per miRNA (restricted to miRNAs with complete data) in Het or KO versus Ctrl, showing widespread loss of target binding per AGO2-miRNA despite increased miRNA expression and AGO2-miRNA enrichment. (**F**) Summed chimeric reads per reproducibly detected AGO2-miRNA:target transcript show dose-dependent reduction in LIMD1-deficient cells (Wilcoxon matched-pairs signed-rank test; ****P < 0.0001). (**G** to **I**) Expanded views of top 25 (left) and next 224 (right) target transcripts show reduced chimeric reads (G), peaks (H), and bound miRNAs (I) per target transcript, ordered by counts in Ctrl. (**J**) Proportional Venn diagrams of selected AGO2-miRNA targetomes show reduced targeting breadth for the top three Ctrl miRNAs (miR-23a-3p, let-7a-5p, and miR-27a-3p). (**K** and **L**) Chimeric reads per matched peak (≥3 reads in each group) for these miRNAs show reduced occupancy per site in LIMD1-deficient cells, extending beyond the targetome losses in (J). (L) Read counts for the top 10 matched interactions labeled per miRNA by target:region:seed-pairing. (**M**) Summed chimeric reads per 3′UTR for transcripts targeted by miR-23a-3p, miR-27a-3p, and let-7a-5p show reduced total reads, target number, and reads per 3′UTR in LIMD1-deficient cells; top 10 Ctrl targets per miRNA are color coded. (**N**) Schematic summarizing reduced targets and target-site occupancy per AGO2-miRNA, and fewer AGO2-miRNAs per target transcript, in LIMD1-deficient cells.

At the level of individual miRNAs, targeting capacity contracted substantially. Median chimeric reads per miRNA were reduced by approximately half, and each AGO2-miRNA engaged fewer binding sites and transcripts (Fig. 5, A to E; and fig. S5, A to D). For example, miR-23a-3p and miR-27a-3p targeted >90 mRNAs in Ctrl cells but <30 in Het or KO cells (Fig. 5D). Correspondingly, binding events per mRNA declined by a median of 48% in Het and 63% in KO, with fewer distinct AGO2-miRNAs per transcript across CDSs and 3′UTRs (Fig. 5, F to I; and fig. S5, I to M).

Analysis of miRNA abundance and AGO2 enrichment supports that this contraction reflects a defect in target engagement rather than reduced miRNA expression or AGO2 loading. Despite the marked fall in chimeric read counts per miRNA, both expression and AGO2-miRNA enrichment of target-engaged miRNAs increased in LIMD1-deficient cells (Fig. 5E and fig. S5D). Absolute miRNA expression did not predict chimeric read output per miRNA (fig. S5E), and changes in miRNA expression did not correlate with changes in per-miRNA chimeric read counts (fig. S5F). By contrast, AGO2-miRNA enrichment correlated positively with chimeric read counts within each genotype (fig. S5G), indicating that more highly AGO2-associated miRNAs generally produced more targeting events. Changes in AGO2-miRNA enrichment also correlated positively, albeit modestly, with changes in chimeric read count (fig. S5H), indicating that greater increases in AGO2 association were associated with comparatively smaller reductions in targeting. This suggests that increased AGO2-miRNA association may partly compensate for reduced targeting capacity in LIMD1-deficient cells, despite an overall decrease in chimeric read counts. However, this potential compensation was insufficient to preserve targeting to near Ctrl levels in LIMD1-deficient cells.

Network visualizations of the three miRNAs with the highest total chimeric reads—miR-23a-3p, let-7a-5p, and miR-27a-3p—provide per-miRNA case studies that illustrate this systematic contraction of targeting, whereby these prevalent AGO2-miRNAs engage fewer targets and exhibit reduced per-site AGO2-miRNA occupancy, reflected by decreased chimeric read counts (Fig. 5, J to M). Together, these transcriptome-wide, miRNA- and mRNA-resolved analyses demonstrate a graded reduction in the breadth and depth of the AGO2-miRNA targeting network in LIMD1-deficient cells (Fig. 5N).

### LIMD1 shapes AGO2-miRNA targeting landscapes by governing motif preferences and positional footprints

**Fig. 6.**
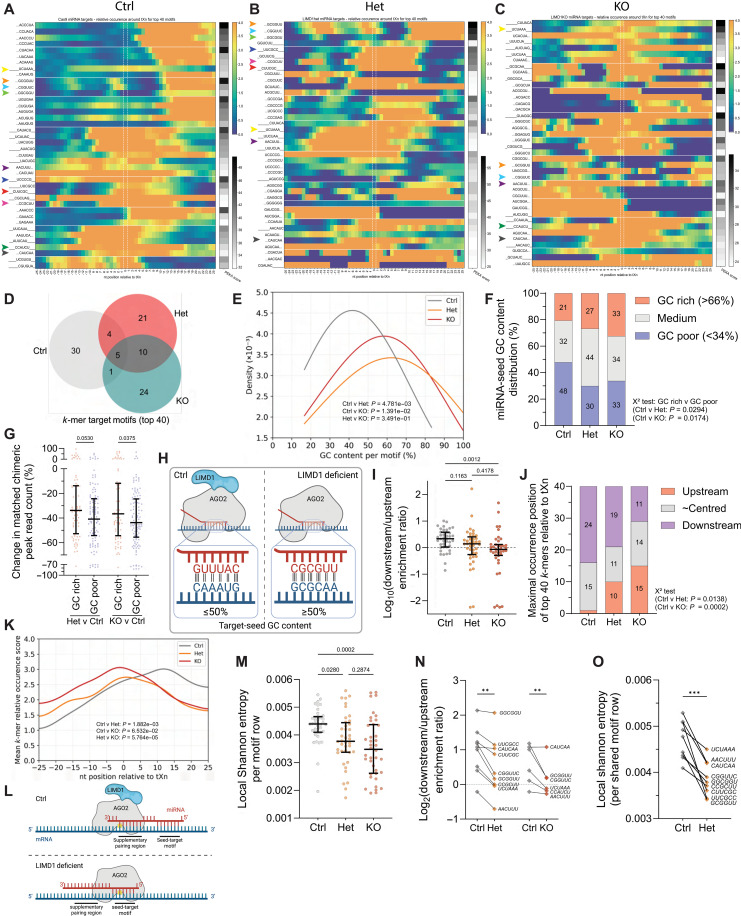
LIMD1 shapes AGO2-miRNA targeting landscapes by governing motif preferences and positional footprints. (**A** to **C**) PEKA of the top 40 target motifs from reproducible chimeric peaks in Ctrl (A), Het (B), and KO (C). Heatmaps depict motif relative occurrence (RtXn), defined as *k*-mer fold enrichment near thresholded cross-link (tXn) sites relative to distal background; PEKA scores reflect enrichment strength and positional consistency. *k*-mers are clustered by sequence similarity and aligned by maximal RtXn; dotted lines indicate cases where maximal enrichment occurs >3 nt from the cross-link site. LIMD1 deficiency alters motif identity, positional distribution, and binding footprints. Colored arrowheads denote matched motifs; enlarged heatmaps are shown in fig. S10 (A to C). (**D**) Venn diagram of top 40 motifs. (**E**) GC content of the top 40 motifs in Ctrl, Het, and KO by Kernel density (corrected Kruskal-Wallis). (**F**) Distribution of GC-rich (>64%), medium, and GC-poor (<34%) canonical seed types in top 40 motifs of each group [chi-square (χ^2^) test]. (**G**) Change in matched chimeric peak read counts for GC-rich and GC-poor canonical seeds in Het and KO versus Ctrl (Kruskal-Wallis). (**H**) Schematic of the typical GC-content shift in canonical seed interactions. (**I** to **K**) Motif-position analyses showing loss of downstream (3′) bias and a dose-dependent upstream (5′), cross-link–proximal shift in LIMD1-deficient cells. (**L**) Schematic summarizing repositioning of peak motif enrichment from downstream of the cross-link site in Ctrl to cross-link–proximal in Het and KO. (**M**) Local Shannon entropy per motif, reflecting reduced footprint flexibility in LIMD1-deficient cells (corrected ANOVA). (**N** and **O**) Shared motifs between Ctrl and Het (*n* = 9) or Ctrl and KO (*n* = 6). Even for identical motifs, LIMD1 deficiency shifts enrichment relatively upstream (N) [corrected mixed effects model using restricted maximum likelihood (REML)] and reduces Shannon entropy (O) (ratio paired *t* test).

The preceding analyses demonstrated that LIMD1 deficiency contracts the AGO2-miRNA targeting network by reducing both the number of engaged targets and the frequency of interaction at individual sites. We therefore asked whether this contraction is accompanied by changes in the sequence features and positional architecture of AGO2-miRNA–bound target sites. To address this, we applied positionally enriched *k*-mer analysis (PEKA) (*42*) to AGO2 chimeric eCLIP target data. PEKA quantifies enriched sequence motifs and their positional distribution relative to thresholded cross-link–centered AGO2 sites, enabling systematic identification of target motifs and their nucleotide-level enrichment around AGO2 binding events. PEKA heatmaps (Fig. 6, A to D) summarize the identity and positional enrichment of the top target-associated *k*-mers in each genotype, allowing direct comparison of motif usage and positional bias relative to the AGO2 cross-link site.

PEKA revealed that LIMD1 deficiency reshapes the target-motif landscape. Of the top 40 *k*-mers identified by PEKA in Ctrl cells, only 9 and 6 were also among the top motifs in Het and KO cells, respectively (Fig. 6, A to D), indicating a marked and progressive reconfiguration of the prevailing motif repertoire associated with AGO2-miRNA targeting. This shift was characterized by a systematic change in motif composition; motifs enriched in LIMD1-deficient cells were consistently more GC-rich (Fig. 6E). Consistent with this PEKA-derived finding, analysis of canonical seed interactions revealed a GC-dependent redistribution, wherein GC-poor motifs were selectively depleted, whereas GC-rich motifs were correspondingly enriched in Het and KO cells (Fig. 6, F to H, and fig. S6A). Because higher GC content confers greater base-pairing stability (*43*), these observations suggest that relatively weaker, GC-poor seed:target interactions may be disproportionately sensitive to LIMD1 loss.

LIMD1 deficiency also altered AGO2’s positional footprint on target mRNAs. In Ctrl cells, target-motif enrichment was biased downstream of the AGO2 cross-link site, as shown by positive downstream/upstream enrichment ratios (Fig. 6I), maximal motif enrichment occurring predominantly downstream of the cross-link site (Fig. 6J), and average enrichment profiles peaking 3′ of the cross-link (Fig. 6K), consistent with models in which AGO2-target contacts lie predominantly upstream of the seed on the mRNA, allowing downstream sequences to mediate 3′ supplementary pairing (*3*, *7*, *44*). In LIMD1-deficient cells, this downstream bias was progressively lost and replaced by a more upstream, cross-link–proximal positional preference (Fig. 6, I to L), consistent with AGO2-miRNA:target interactions becoming increasingly constrained around seed-proximal contacts. Figure 6L is an interpretive summary of these positional shifts, capturing the ∼14-nucleotide (nt) change measured in Fig. 6K, from a peak ∼12 nt downstream of the cross-link in Ctrl to a cross-link–proximal peak (−2 nt) in KO. This is consistent with AGO2-target contacts shifting from upstream of the seed in Ctrl to more seed-proximal contacts in LIMD1-deficient cells, with loss of upstream contacts that may support supplementary pairing.

To further characterize AGO2’s positional footprint, we analyzed motif enrichment profiles and found evidence of altered interaction architecture. In Ctrl cells, enrichment profiles included a broad distribution of moderately enriched nucleotide positions, whereas LIMD1-deficient cells exhibited a more bimodal distribution, characterized by an increase in zero-occurrence sites and a loss of intermediate-enrichment positions (Fig. 6, A to C, and fig. S6B). To quantify this, we calculated local Shannon entropy across enrichment profiles as a measure of positional variability. Ctrl cells exhibited higher entropy, indicating smoother enrichment gradients and broader, more flexible footprints, whereas Het and KO cells showed lower entropy and more punctate profiles (Fig. 6M). Consistent with this, reproducible chimeric peak widths were modestly but significantly narrower in LIMD1-deficient cells, providing an independent measure of reduced AGO2 footprint breadth at target sites (fig. S6C).

Because these positional shifts could be confounded by the observed changes in motif identity, we performed shared-motif analyses restricted to sequences present across genotypes. This controlled for intrinsic binding geometries of specific *k*-mers and revealed that even identical motifs showed a consistent upstream positional shift and reduced entropy in LIMD1-deficient cells (Fig. 6, N and O; and fig. S6, D to G). These results indicate that LIMD1 affects AGO2 positional preference and footprint flexibility independently of motif identity, consistent with LIMD1 stabilizing a broader and more permissive miRISC:target interaction architecture.

Together, these findings indicate that LIMD1 shapes the AGO2-miRNA targeting landscape at two levels: by influencing which motifs are engaged and by modulating the positional footprint of AGO2 on target RNAs. In the absence of LIMD1, the targeting repertoire reconfigures toward high-stability motifs and exhibits more restricted positional footprints. This transition is consistent with reduced tolerance for weaker seed interactions and may contribute to the contraction in miRNA regulatory breadth observed in LIMD1-deficient cells.

### LIMD1 deficiency derepresses miRNA targets in hSAECs and LUAD

**Fig. 7.**
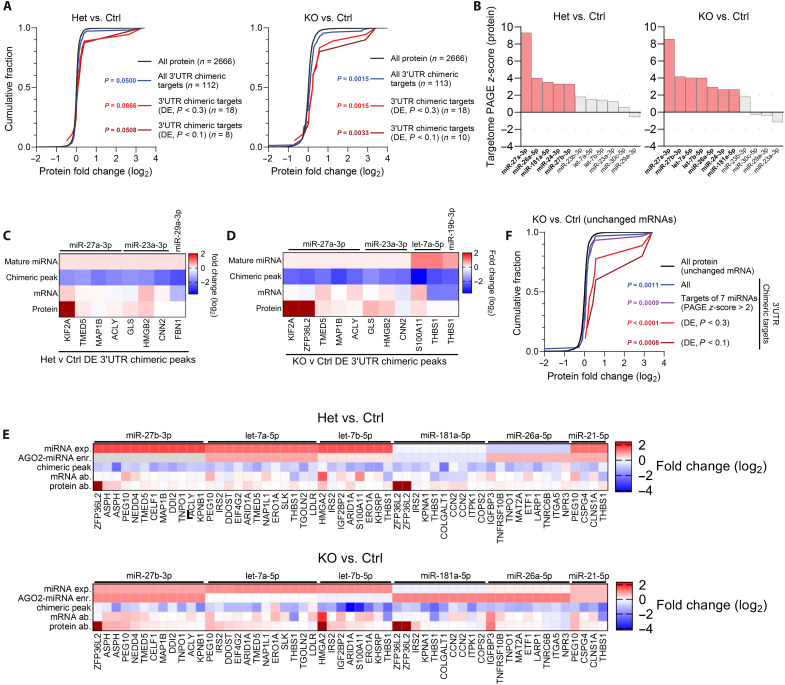
LIMD1 deficiency derepresses miRNA targets in hSAECs. (**A**) Cumulative distribution of protein fold changes (log_2_) in Het or KO versus Ctrl, measured by MS from six independent replicates of Ctrl C4, Het C6, and KO C2 hSAECs. Proteins with 3′UTR chimeric peaks (blue) show increased abundance relative to all proteins (black), strongest for the most de-enriched peaks [false discovery rate (FDR) < 0.3, red; FDR < 0.1, dark red; multiplicity-corrected Kruskal-Wallis test]. (**B**) PAGE *z*-scores of AGO2-miRNA targetomes show increased protein levels of chimeric targets; significant increases (*z* > 1.96 ≡ *P* < 0.05) are highlighted in red. (**C** to **E**) Heatmaps of log_2_ fold changes in miRNA expression, AGO2-miRNA enrichment, chimeric reads, mRNA abundance, and protein abundance (restricted to targets with complete data, including protein). (C and D) De-enriched 3′UTR chimeric targets (FDR < 0.1) in Het (C) and KO (D). (E) Full 3′UTR chimeric targetomes of selected miRNAs, showing consistent patterns across datasets (gray, missing data). Color scale capped to enhance visibility of submaximal protein increases; log_2_ change > 3 shown as dark red. Repeated gene names correspond to distinct chimeric peaks mapping to the same transcript. (**F**) Cumulative distribution of protein fold changes in KO versus Ctrl for proteins with unchanged mRNA abundance (0.8- to 1.2-fold). Protein levels are increased for 3′UTR chimeric targets (blue), targets of AGO2-miRNAs with PAGE *z*-scores of >2 (purple), and de-enriched chimeric targets (red/dark red), consistent with translational derepression independent of mRNA decay (multiplicity-corrected Kruskal-Wallis test).

Given the widespread loss of AGO2-miRNA:target interactions in LIMD1-deficient cells, we next asked whether miRNA targets were correspondingly derepressed. Quantitative proteomics revealed a net relative increase in protein abundance for transcripts harboring 3′UTR chimeric sites, a shift that scaled with both the degree of LIMD1 loss and the extent of chimeric peak depletion (Fig. 7A and fig. S7A). To determine whether this shift reflected coordinated changes across miRNA target sets, we performed parametric analysis of gene expression (PAGE) (*45*). AGO2-miRNA targets defined by either conventional or chimeric eCLIP showed elevated protein expression, with the most strongly de-enriched 3′UTR chimeric sites yielding the highest *z*-scores (fig. S7B).

PAGE further revealed derepression at the level of entire AGO2-miRNA targetomes. Across 11 chimeric-eCLIP–defined target sets, five showed increased protein abundance in Het cells and seven in KO cells (Fig. 7B and fig. S7, C and D). Consistently, proteins encoded by transcripts with de-enriched chimeric interactions (*P* < 0.1), as well as those belonging to entire AGO2-miRNA targetomes, were elevated despite increased cognate miRNA abundance and AGO2-miRNA enrichment (Fig. 7, C to E, and fig. S7E). Furthermore, increased protein levels for stable or up-regulated transcripts indicate that LIMD1 deficiency impairs translational repression in addition to mRNA decay (Fig. 7F and fig. S7F).

**Fig. 8.**
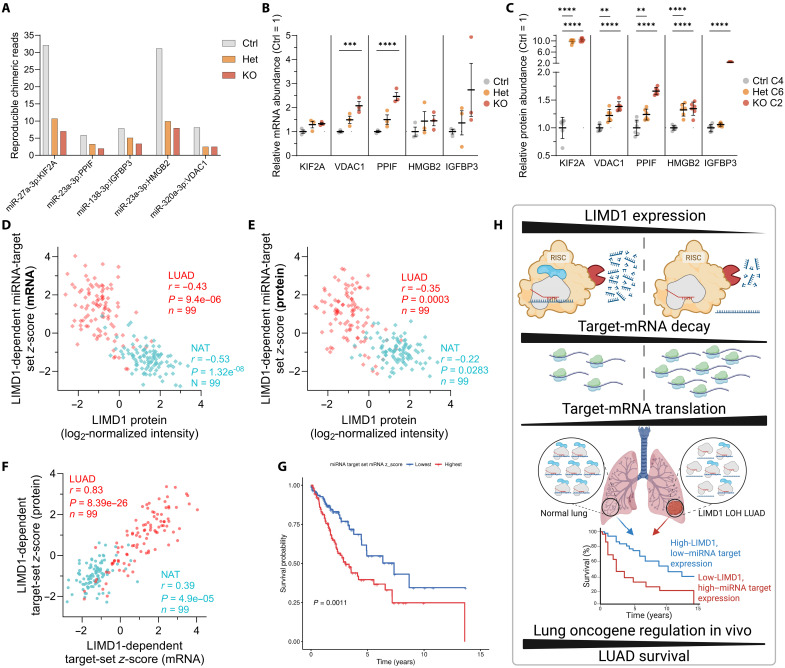
LIMD1-dependent targets inversely correlate with LIMD1 in normal and LUAD lung tissue and their dysregulation predicts LUAD survival. (**A** to **C**) A five-gene target set (*KIF2A*, *VDAC1*, *PPIF*, *HMGB2*, and *IGFBP3*) was identified as LIMD1-dependent in hSAECs for subsequent analysis, together with LIMD1, in public lung datasets. Genes were selected based on (A) reduced chimeric reads per reproducible peak, (B) increased mRNA abundance by mRNA-seq, displayed as normalized read counts with DESeq2-adjusted *P* values (***P < 0.001; ****P < 0.0001), and (C) increased protein abundance by MS with FDR-corrected *P* values (**, FDR < 0.01; ****, FDR < 0.0001), in Het and KO relative to Ctrl. For (B) and (C), data are normalized to a Ctrl mean of 1. (B) Three CRISPR biological replicates (Ctrl C2/C3/C4), and (C) six independent MS replicates per genotype using Ctrl C4, Het C6, and KO C2 samples. (**D** and **E**) In CPTAC LUAD samples, LIMD1 protein levels inversely correlate (Pearson) with target-set *z*-scores at mRNA (D) and protein (E) levels for five de-enriched 3′UTR targets up-regulated in LIMD1-deficient hSAECs. *r*, correlation coefficient. (**F**) Correlation between target-set mRNA and protein *z*-scores is stronger in LUAD than NAT, consistent with loss of translational control in tumors. (**G**) Kaplan-Meier survival analysis of patients with LUAD (TCGA-LUAD) stratified by target-set mRNA signature: Patients in the highest quartile (red) had poorer survival than those in the lowest quartile (blue; log rank, *P* = 0.0011; *n* = 129 per group). (**H**) Schematic illustrating LIMD1-dependent regulation of target mRNA decay and translation; LIMD1 protein correlates with in vivo miRNA:target-oncogene expression; low LIMD1 and high miRNA:target-oncogene expression associates with poorer LUAD survival.

We next tested whether these effects extend beyond CRISPR-engineered hSAECs and into normal and diseased lung tissue. To test whether the molecular consequences of LIMD1-deficiency extend beyond engineered hSAECs, we defined a five-gene LIMD1-dependent target signature in our hSAEC models (*KIF2A*, *VDAC1*, *PPIF*, *HMGB2*, and *IGFBP3*; Fig. 8, A to C) and then examined this signature, together with *LIMD1* mRNA and LIMD1 protein levels, in public LUAD datasets. Without selecting for these traits, we note that four of five have published oncogenic roles in LUAD, and three (*VDAC1*, *HMGB2*, and *KIF2A*) have been directly implicated in miRNA-associated dysregulation in NSCLC (*46*–*52*). In LUAD tumors, LIMD1 protein was markedly lower than in matched normal adjacent tissue (NAT), as expected given the frequent LIMD1 deficiency arising from LOH reported previously (Fig. 8, D and E; and fig. S8, D and E) (*30*, *31*, *35*). In parallel, both mRNA and protein abundance of the target set were elevated in LUAD relative to NAT; notably, target-set mRNA and protein levels inversely correlated with LIMD1 protein in both LUAD tumors and matched NAT (Fig. 8, D and E; and fig. S8, D and E). Moreover, the target set showed stronger mRNA-protein concordance (Fig. 8F), consistent with reduced translational control in LIMD1-deficient LUAD (*53*, *54*).

Clinically, high mRNA levels of this target set predicted poor patient survival and remained a significant prognostic factor in multivariate models; a composite score incorporating LIMD1 and target-set mRNA abundance provided stronger patient stratification (Fig. 8G and fig. S8, F to J). Together, these findings support a model in which LIMD1 protein abundance modulates expression of miRNA targets in human lung tissue, establishing a link between prevalent LIMD1 deficiency, impaired miRNA-mediated repression, and adverse clinical outcomes in LUAD (Fig. 8H).

### LIMD1 augments evolutionarily young yet thermodynamically strong interactions, enriched in C_2_H_2_-zinc-finger genes

LIMD1 is a metazoan-specific adaptor protein within the Zyxin family, a lineage that diversified with the emergence of multicellular complexity (*32*). Given the profound disruption of AGO2-miRNA targeting in LIMD1-deficient cells, together with the capacity of LIMD1 incorporation into miRISC to increase regulatory complexity and the observation that some interactions appear more sensitive to LIMD1 loss than others, we asked whether LIMD1 preferentially supports recently evolved regulatory circuits, thereby contributing to the evolutionary expansion of miRNA networks.

**Fig. 9.**
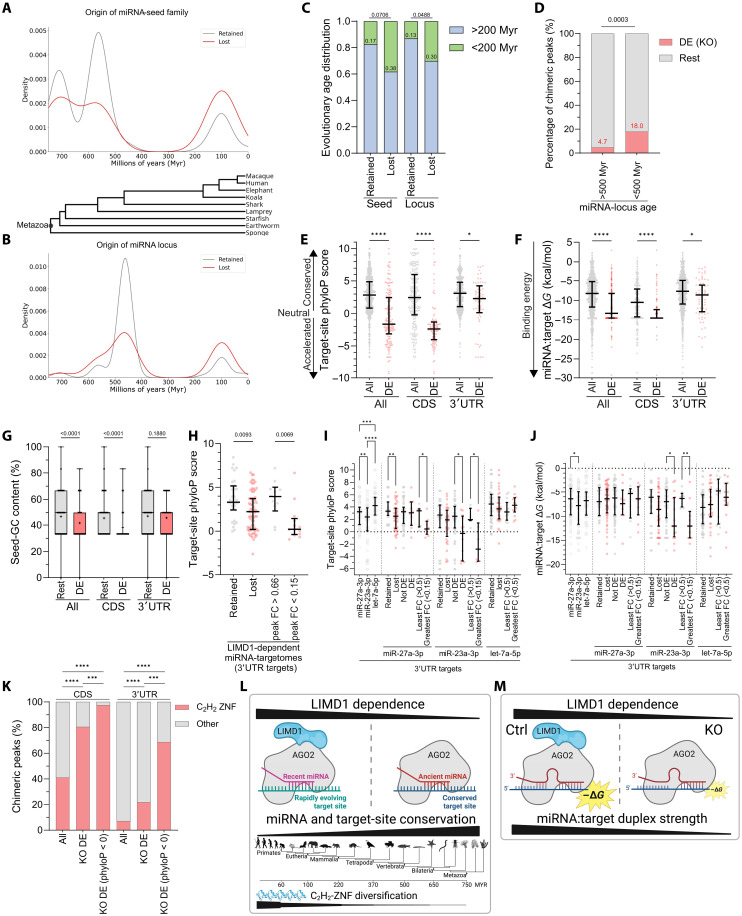
LIMD1 augments evolutionarily young yet thermodynamically strong interactions. (**A** and **B**) Kernel density distributions of evolutionary ages for miRNA seed families (A) and loci (B) reproducibly detected in chimeric peaks (“target-engaged miRNAs”). Families and loci retaining target binding in KO (gray) trace to older origins, whereas those losing target binding in KO (red) are enriched in mammalian-specific lineages; the phylogenetic tree provides context. (**C**) A higher proportion of younger [<200 million years (Myr)] than older (>200 Myr) miRNAs lose target binding in KO (Fisher’s exact test). (**D**) LIMD1-dependent AGO2-miRNA:target interactions [depleted (DE) in KO, *P* < 0.3] are disproportionately associated with younger miRNAs (<500 Myr; Fisher’s exact test). (**E** to **G**) LIMD1-dependent canonical interactions (DE peaks in KO, *P* < 0.3) exhibit lower site conservation (E), greater duplex stability (F), and lower seed GC content (G) than overall CDS/3′UTR targets (corrected Kruskal-Wallis). PhyloP scores of >0 indicate increasing conservation, whereas scores of <0 indicate accelerated evolution; duplex stability was assessed by minimum free energy (Δ*G*), where increasingly negative values reflect stronger base pairing. (**H**) For selected LIMD1-dependent miRNAs (miR-27a-3p, miR-27b-3p, miR-26a-5p, miR-24-3p, and miR-181a-5p; Fig. 7B), sites lost or strongly depleted in KO are less conserved than retained or minimally depleted sites (corrected Kruskal-Wallis). (**I** and **J**) Canonical 3′UTR peaks of miR-27a-3p, miR-23a-3p, and let-7a-5p stratified by LIMD1 dependence show that LIMD1-dependent sites for miR-27a-3p and miR-23a-3p have lower phyloP scores, and for miR-23a-3p also more stable duplexes (more negative Δ*G*; right). Only *P* < 0.05 is shown. (**K**) LIMD1-dependent target sites are strongly enriched for C_2_H_2_ zinc-finger (*ZNF*) genes, especially in CDS (87 of 108, 81%), and even more so among accelerated sites (phyloP < 0) (HUGO Gene Nomenclature Committee categories; chi-square test). (**L** and **M**) Schematics summarizing that the most LIMD1-dependent interactions involve younger miRNAs or less conserved target sites and form stronger AGO2-miRNA:target duplexes.

We first tested whether AGO2-miRNAs that retained target binding in LIMD1 KO cells (≥1 reproducible chimeric peak in Ctrl and KO) were more evolutionarily conserved than those that lost target binding entirely (no reproducible peak in KO). Those retaining target-binding traced largely to bilaterian seed families and to loci arising from vertebrate whole-genome duplications, whereas those that lost binding were enriched for mammalian-specific seed families and loci (*29*); evolutionary conservation independently predicted retention of target-binding in KO (Fig. 9, A to C; and fig. S9, A to C). Moreover, LIMD1-dependent interactions, defined as 3′UTR chimeric peaks depleted in KO cells, were disproportionately associated with younger miRNAs (Fig. 9D). Together, these observations suggest that LIMD1 preferentially supports interactions involving evolutionarily younger miRNAs, potentially extending the regulatory reach of AGO2-miRNA networks.

To further examine sequence features associated with LIMD1 dependence, we analyzed canonical target sites using phyloP conservation scores (*55*) to assess evolutionary constraint and predicted hybridization energies of 22-nt miRNA:target duplexes (*54*), as a measure of interaction thermodynamic stability. Analyses were stratified by target region, interaction class, and degree of LIMD1 dependence (fig. S9, D to F). This framework allowed us to examine how target-site evolutionary conservation and duplex stability relate to differential sensitivity of AGO2-miRNA:target interactions to LIMD1 loss.

LIMD1 dependence (−log_10_ *P* value) correlated with lower site conservation, reduced seed GC content, and stronger predicted duplexes, particularly within CDS sites (fig. S9E), and LIMD1-dependent sites were enriched for these features (Fig. 9, E to G). These relationships persisted among transcripts with unchanged or increased abundance (fig. S9G). Within 3′UTR targetomes of LIMD1-dependent miRNAs, the most depleted sites were consistently less evolutionarily conserved (Fig. 9H and fig. S9H).

These trends extended to the canonical 3′UTR target repertoires of individual miRNAs, with variability in LIMD1 dependence evident both between miRNAs and within the canonical repertoire of a single miRNA. For example, within the miR-23a-3p 3′UTR canonical repertoire, less conserved sites predicted to form more stable duplexes showed greater LIMD1 dependence, whereas the more ancient let-7a-5p repertoire did not exhibit LIMD1 dependence based on these features (Fig. 9, I and J). These observations suggest that canonical seed pairing alone may be insufficient for AGO2-miRNA engagement at specific loci and that efficient targeting at a subset of sites defined by underlying RNA features may depend disproportionately on LIMD1.

The most LIMD1-dependent sites were predominantly low-conservation, highly stable 8-mer interactions enriched in C_2_H_2_-zinc-finger (*C*_*2*_*H*_*2*_*-ZNF*) transcripts (Fig. 9K and fig. S9, I to L), a transcription factor family that has undergone extensive lineage-specific expansion, particularly in primates (*56*–*58*). Many of these sites exhibited negative phyloP scores, consistent with accelerated sequence evolution, a recognized feature of regulatory elements within this rapidly evolving gene family prone to lineage-specific and clade-restricted diversification (*56*, *57*).

Collectively, these findings suggest that, beyond broadly augmenting miRNA-mediated repression, LIMD1 dependence is most pronounced for interactions involving evolutionarily younger miRNAs and less conserved target sites predicted to form thermodynamically stable duplexes, particularly within CDSs and *C*_*2*_*H*_*2*_*-ZNF* transcripts (Fig. 9, L and M). These patterns indicate that LIMD1 dependence is shaped by underlying RNA features that influence AGO2-miRNA target engagement.

**Fig. 10.**
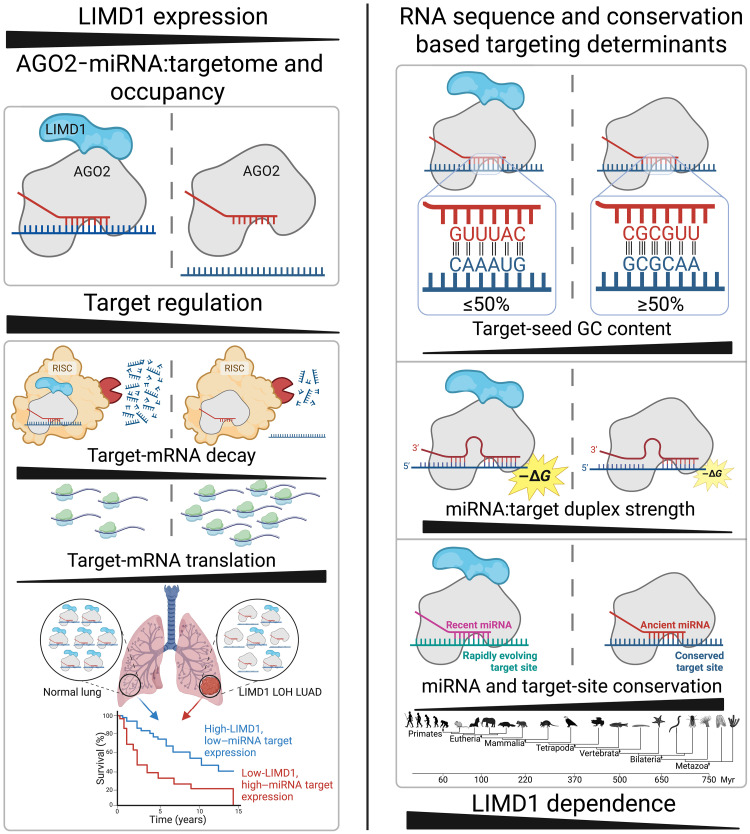
LIMD1 promotes productive AGO2-miRNA targeting and expands the functional and evolutionary reach of miRNA repression. Schematic summary of the model supported by this study. (**Left**) LIMD1 promotes productive AGO2-miRNA target engagement, increasing targetome breadth, site occupancy, target-mRNA decay, and translational repression; LIMD1 deficiency contracts AGO2-miRNA targeting and increases protein abundance from transcripts with LIMD1-sensitive miRNA target sites. In lung cancer, reduced LIMD1 expression is associated with increased expression of LIMD1-dependent miRNA targets and poorer outcome. (**Right**) LIMD1 dependence is greatest for GC-poor seed interactions, thermodynamically strong miRNA:target duplexes, and younger, less-conserved miRNAs and target sites.

Together, the data support a model in which LIMD1 promotes productive AGO2-miRNA targeting by expanding targeting breadth, target-site occupancy, repression, and the evolutionary reach of endogenous miRNA interactions (Fig. 10).

## DISCUSSION

Our findings substantially extend a previously defined LIMD1-AGO2 interaction framework (*19*), implicating LIMD1 as an AGO2 adaptor-scaffold that augments AGO2-miRNA target engagement and repression across the transcriptome. In LIMD1-deficient cells, AGO2 associates with more miRNAs yet engages far fewer transcripts and sites. Individual targetome analyses showed that each AGO2-miRNA bound fewer targets, with reduced occupancy at remaining sites, whereas target- and site-level analyses revealed fewer AGO2-miRNAs engaged per target site and reduced binding across entire mRNAs. This uncoupling of AGO2-miRNA association from productive downstream target engagement is accompanied by widespread protein derepression that scales with LIMD1 loss, consistent with impaired miRNA-mediated repression through both translational control and mRNA decay. These findings support a model in which miRNA targeting outcomes are not dictated by miRNA abundance or pairing rules alone but are also shaped by dynamic miRISC composition, with adaptor-scaffold incorporation providing an additional determinant of AGO2-miRNA target engagement beyond miRNA abundance, AGO2 loading, or sequence features (*1*, *8*, *27*).

Notably, LIMD1 dependence varies even among canonical seed-matched sites within the repertoire of a single miRNA, indicating that, while seed-pairing remains necessary for canonical targeting, it is not necessarily sufficient to predict whether productive AGO2 engagement and repression will occur in cells; adaptor presence may commonly be required for stable engagement. Thus, we propose that LIMD1 modifies the outcome of sequence-defined interactions that would otherwise be infrequent, unstable, or fail to mature into productive repression. Elegant in vitro studies have been invaluable in defining quantitative rules linking site sequence, structure, and affinity to AGO-miRNA target binding and repression, but these largely RNA-centric frameworks do not fully capture the contribution of AGO-associated protein partners or higher-order AGO-containing assemblies. Biochemical reconstitution has shown that multivalent TNRC6 binding can drive cooperative AGO assembly on target RNAs (*22*), marking an important advance in defining how protein partners shape target engagement. However, higher-order miRISC assemblies remain difficult to copurify and interrogate, and endogenous targeting may depend on dynamic assembly states not fully recapitulated in transformed cell systems (*14*, *36*), leaving broader adaptor contributions underexplored. Together, our findings may help explain why, despite recent advances, in vivo miRNA:target prediction remains incomplete and support an extension of RNA-centric models of miRNA function to include miRISC scaffold composition as an additional determinant of targeting efficacy (*8*, *27*). Accordingly, predictive models and small-RNA therapies should consider advancing beyond sequence features alone to better account for miRISC architecture (*8*, *59*, *60*).

Mechanistically, our data are consistent with LIMD1 functioning as a miRISC adaptor-scaffold that broadens AGO2’s targetome and sustains AGO2-miRNA:target interactions across both high- and low-occupancy sites, which contract in its absence. LIMD1 deficiency shifts AGO2-miRNA targeting toward narrower binding contexts: GC-poor seed motifs are disproportionately depleted, with the remaining targeting landscape biased toward GC-rich, higher-affinity seed interactions (*43*) and AGO2 footprints shifted to more seed-proximal, constrained profiles (*3*, *44*, *61*, *62*). Paradoxically, canonical interactions predicted to form highly stable miRNA:mRNA duplexes often exhibit greater LIMD1 dependence, indicating that final duplex stability alone is not sufficient to explain productive engagement in cells. This is consistent with evidence that target-site accessibility and the energetic cost of unpairing local mRNA structure can be as important for repression as seed complementarity itself, and that effective targeting may also require local flanking sequence to become accessible to the RISC-bound miRNA (*8*, *63*, *64*).

A plausible basis for this selectivity is suggested by LIMD1’s mapped interfaces within miRISC. The AGO-binding motif in the pre-LIM region of LIMD1 engages AGO2 at the L2 region, while the C-terminal LIM domains bind the TNRC6A N terminus (*19*), positioning LIMD1 as a composite scaffold bridging AGO2 to TNRC6A through separable interfaces. Structural work indicates that AGO2 presents the guide seed in a preorganized binding chamber, while the guide central and 3′ regions remain comparatively dynamic and are reorganized upon target binding; access to nucleotides beyond the seed is controlled by a central gate within the RNA-binding cleft, to which the L2 and PIWI loops contribute (*3*, *15*, *23*, *24*, *65*). In this context, LIMD1 binding at the AGO2 L2 region could plausibly influence the conformational gating that determines whether an initial seed-paired encounter is stabilized sufficiently to progress to productive target engagement rather than dissociation.

This framework suggests two nonmutually exclusive interpretations of our sequence and footprint data. First, weak, often GC-poor, seeds may be intrinsically less stable at the initial seed-pairing step and, therefore, more likely to dissociate unless additional stabilization is provided. Second, weaker seed pairing may permit greater conformational flexibility or lateral sampling along the target, increasing the chance of reaching a more stable duplex state via supplementary pairing if the complex persists long enough. In either case, an AGO2 adaptor that stabilizes seed-paired intermediates, prolongs effective dwell time, or biases AGO2 toward productive target-bound conformations would be expected to disproportionately affect such interactions. These possibilities are not mutually exclusive with a role for target accessibility: Preexisting mRNA secondary structure may make initial seed engagement energetically unfavorable, while efficient 3′ pairing can compensate for low seed accessibility and rescue otherwise ineffective sites (*64*–*67*). Recent work further suggests that AGO2 itself can compress the affinity range of RNA:RNA interactions, strengthening weaker binders while attenuating stronger ones (*27*), implying that adaptor-mediated effects on AGO2 conformation or assembly state may be especially consequential at structurally occluded or otherwise marginal sites.

Supplementary pairing provides one plausible route by which this could occur. In AGO2, formation of the supplementary chamber requires repositioning of the L2 and PIWI loops together with movement of the L2/helix-7/PAZ module to permit pairing at g13–g16 (*3*, *15*, *23*, *24*, *65*). LIMD1 binding at AGO2 L2 could, therefore, in principle, favor progression from initial seed recognition to broader target sampling and supplementary pairing, while its bridging of AGO2 to TNRC6 proteins could help stabilize these more productive assemblies once formed. We therefore speculate that LIMD1-AGO2 interaction may increase the probability that transient AGO2-miRNA:target encounters mature into silencing-competent complexes, helping miRISC overcome kinetic or structural barriers in vivo and explaining why GC-poor seeds and other kinetically challenging sites show disproportionate LIMD1 dependence; GC-rich seeds may more readily stabilize early intermediates and thus require less adaptor-mediated support.

Whether LIMD1 primarily acts through its direct interaction with AGO2 (which is not necessarily contingent on TNRC6A) (*19*), through bridging AGO2 to TNRC6 proteins to stabilize and/or remodel AGO2-TNRC6 assemblies, or through a combination of both remains unresolved. Separating the contributions of these interfaces and their order of action during target recognition and repression will be important for defining how AGO adaptors influence AGO-miRNA:target structure and functional outcome. Additional limitations include incomplete paralog coverage (AGO/TNRC6); reliance on immortalized hSAECs; chimeric-eCLIP–intrinsic biases; the lack of direct biochemical, structural, or kinetic tests of the proposed dwell-time, conformational-gating, or assembly-state models; the absence of experimental dissection of why AGO2-miRNA association increases in LIMD1-deficient cells; and the lack of direct analysis of how miRISC assembly states differ in LIMD1-deficient cells and their relationship to distinct targeting profiles. Addressing these limitations will require targeted biochemical, structural, and kinetic studies. Nonetheless, LIMD1 deficiency reproducibly compromises AGO2-miRNA target engagement and repression, supporting a broader view in which AGO-associated adaptor proteins can shape the effectiveness and topology of functional miRNA regulatory networks.

The implications may extend beyond cell culture. *LIMD1* LOH is frequently clonal in NSCLC and associates with reduced expression and poorer survival (*30*, *31*, *35*). In lung tissue datasets, *LIMD1* mRNA and LIMD1 protein levels inversely correlate with a miRNA-target signature that is elevated in tumors relative to matched NAT and is prognostic for LUAD survival. Together with in vivo evidence that miRNA-mediated repression constrains alveolar stem cell state transitions and oncogenic programs in the lung (*68*), these data implicate LIMD1 as an important determinant of miRNA network integrity in this tissue. *LIMD1* haploinsufficiency through LOH may therefore contribute early to widespread target dysregulation via impaired miRNA-mediated repression and, in turn, be associated with adverse outcome, although prospective validation of this model will be required.

Our evolutionary analyses suggest that LIMD1 dependence is enriched among interactions involving younger, mammalian-specific miRNAs and less conserved target sites; these sites are more often associated with signatures consistent with accelerated evolution, are predicted to form stable duplexes, and are enriched within *C*_*2*_*H*_*2*_*-ZNF* mRNAs, particularly in CDSs. Although the timing and evolutionary trajectory of LIMD1 co-option into miRISC remains unknown, these patterns raise the possibility that adaptor-governed miRISC architecture can facilitate engagement at newly emerged or rapidly evolving sites, extending AGO2-miRNA targeting beyond deeply conserved interactions. Consistent with this, LIMD1 dependence varies within individual miRNA repertoires, with sites showing heightened sensitivity tending to be less conserved, suggesting that adaptor context can tune the effective regulatory reach of even conserved miRNAs toward newly evolved targets. The pronounced LIMD1 dependence observed at accelerated sites within *C*_*2*_*H*_*2*_*-ZNFs*, a transcription factor family that expanded in tetrapods and diversified extensively in mammals, particularly in primates (*56*, *57*, *69*, *70*), highlights a potential link between adaptor-mediated miRISC function and the evolution of regulatory complexity. More broadly, our findings support a model in which adaptor proteins may contribute to the diversification of posttranscriptional regulation in higher eukaryotes by tuning which sequence-defined interactions mature into functional repression.

In sum, our work identifies an adaptor-governed layer of AGO2-miRNA–mediated regulation in which LIMD1-dependent miRISC context helps determine whether sequence-defined interactions resolve into productive repression. By extending a defined LIMD1-AGO2-TNRC6 framework to endogenous AGO2-miRNA targeting at scale, we show that adaptor biology can modulate the reach and efficacy of miRNA networks, pointing to miRISC adaptor context as an additional determinant of targeting outcome. At a time when existing prediction frameworks still explain only approximately half of the variability in miRNA-mediated repression (*8*), we highlight a testable frontier: how AGO adaptors integrate with RNA pairing rules and features to specify and potentiate target selection and repression in vivo.

## MATERIALS AND METHODS

### Cell culture

Primary-derived hSAECs (Lonza, CC2547) were maintained in hSAEC basal growth medium with supplements (Sigma-Aldrich, C-21170) at 37°C in 5% CO_2_ without antibiotics. Medium was refreshed every 2 to 3 days. At ∼70 to 80% confluence, cells were washed in phosphate-buffered saline (PBS), detached with Trypsin/ethylenediaminetetraacetic acid (EDTA; Gibco, R001100) for ∼3 min, neutralized with DMEM (Sigma-Aldrich, D8437) containing 10% fetal bovine serum (Gibco, 10270106), pelleted at 300*g* for 3 min, and resuspended in fresh hSAEC medium. Cell counts were determined using a Countess II Automated Cell Counter (Invitrogen), and all cultures were routinely screened for mycoplasma. Wild-type (WT) hSAECs were semi-immortalized by pFLRu-Bmi-1 transduction and subsequent puromycin selection.

### CRISPR-Cas9 *LIMD1* editing and validation

To create *LIMD1* Het and KO hSAECs, WT hSAECs were edited using the Dharmacon Edit-R CRISPR-Cas9 platform (Horizon Discovery), comprising enhanced green fluorescent protein–Cas9 mRNA (CAS11860), Edit-R trans-activating CRISPR RNA (tracrRNA; U-002005), and a predesigned CRISPR RNA (crRNA) targeting exon 1 of *LIMD1* (guide #3; 5′-CCGAGTTTGAGGAAACTCGC-3′). A nontargeting synthetic sgRNA (#2; 5′-proprietary sequence-3′; U-007502-01-05) served as negative control (to create Ctrl hSAECs). Transfections were performed with DharmaFECT Duo (Horizon, T-2010) according to the manufacturer’s protocol. Twenty-four hours after transfection, cells were seeded at limiting dilution (one cell per well) into 96-well plates for single-cell clonal expansion. Colonies were expanded after 2 weeks and transferred to replicate plates, where individual clones were screened by immunoblotting to identify potential Het and KO genotypes alongside controls (Ctrl).

*LIMD1* mutations in candidate Het and KO clones were verified by polymerase chain reaction (PCR) amplification of the targeted exon 1 locus, followed by Sanger sequencing (SourceBioscience). PCR products were generated with Phusion HF master mix [New England Biolabs (NEB), M0531S] and primers 5′-GAGTAGAGGCCCTGTCAATGG-3′ (forward) and 5′-CACAGATCCCAGGCTACCATC-3′ (reverse), purified, and sequenced using universal T7/SP6 primers.

Whole genome sequencing was subsequently performed to confirm on-target editing and to assess potential off-target effects that could impact miRNA-mediated regulation. Genomic DNA was extracted, quantified (NanoDrop OD260/280 = 1.8 to 2.0), and libraries were prepared by Novogene with ∼350–base pair (bp) inserts, Illumina adapters, and limited-cycle PCR enrichment. Sequencing was performed on an Illumina NovaSeq 6000 with 150-bp paired-end reads, targeting ∼30× mean coverage across the GRCh38 reference genome. Reads were filtered for quality and aligned with Burrows-Wheeler Aligner maximal exact matches (BWA-MEM), duplicates were marked with Picard, and variants were called using Genome Analysis Toolkit (GATK) HaplotypeCaller. Putative off-target events were identified by local alignment of all detected variants against the 23-nt guide sequence plus protospacer adjacent motif (PAM), requiring ≥13-nt contiguous seed match, ≤3 mismatches, and ≥10 supporting reads. No high-confidence off-target edits were detected.

### Western blotting

Cells were lysed in radioimmunoprecipitation assay buffer [150 mM NaCl, 1% (v/v) IGEPAL, 0.5% (w/v) deoxycholic acid, 0.1% (w/v) SDS, and 50 mM tris-HCl (pH 7.5)] supplemented with protease (Pierce, A32963) and phosphatase inhibitors (Roche, 12352204) during the log phase of growth. Protein concentration was quantified using the Pierce bicinchoninic acid (BCA) Protein Assay Kit (Thermo Fisher Scientific, 23225). Equal amounts of protein lysate (15 to 30 μg per lane) were resolved by SDS–polyacrylamide gel electrophoresis, transferred onto polyvinylidene difluoride membranes, and probed with the following primary antibodies: mouse anti-LIMD1 (in-house, clone 3F2 C6), mouse anti–β-actin (Sigma-Aldrich, A1978), mouse anti-vinculin (Invitrogen, 14-9777-82), rabbit anti-AGO2 (Sino Biological, 50683-RP02), rabbit anti-GW182/TNRC6A (Bethyl, A302-329A), and rabbit anti-TNRC6B (Sigma-Aldrich, AB9913). Horseradish peroxidase–conjugated secondary antibodies (Dako, P0214) were used in combination with enhanced chemiluminescence reagents (Thermo Fisher Scientific; Millipore, WBKLS0500). Blots were imaged using a GE Healthcare ImageQuant system. Densitometry of immunoblot band intensity was performed in ImageJ, with target signal intensities normalized to the vinculin loading control.

### Coimmunoprecipitation

Cells from a 15-cm dish (one dish per elution) were lysed in 1 ml of NP-40 buffer [150 mM NaCl, 50 mM tris-HCl (pH 8.0), 0.7% NP-40, and 5% glycerol] supplemented with protease and phosphatase inhibitors. Lysates were clarified by centrifugation at 14,000*g* for 5 min at 4°C. One milliliter (∼0.5 mg of total protein) of cleared lysate was incubated with 1 μg of antibody [mouse immunoglobulin A (IgA) isotype control (eBioscience/Invitrogen, 14-4762-81) or mouse IgA anti-AGO2 (Santa Cruz Biotechnology, sc-53521)] overnight at 4°C with rotation. For inputs, 80 μl of cleared lysate was mixed with 20 μl of 5× Laemmli buffer and boiled for 5 min. Antibody-lysate mixtures were washed twice in 500 μl of NP-40 buffer and resuspended in 30 μl of NP-40 buffer. Protein L magnetic beads (Pierce, 88849) were then added and incubated for 1 hour at room temperature with rotation to capture antibody-protein complexes. Beads were washed four times in 1 ml of PBS containing 0.1% Tween 20. Protein complexes were eluted by addition of 40 μl of 0.2 M glycine (pH 2.5) with 3 min of incubation at room temperature, with regular mixing. Eluates were transferred to new tubes and neutralized with 5 μl of 1 M tris-HCl (pH 8.0). Following addition of 11.25 μl of 5× Laemmli buffer and boiling for 5 min, samples were analyzed by Western blotting as described above. Densitometry was performed in ImageJ, with Co-IP band intensities normalized to the relative input control (AGO2).

### PLA and analysis

hSAECs grown on chamber slides (Nunc Lab-Tek II CC2 Chamber Slide System; Thermo Fisher Scientific, 154852) were fixed with 4% paraformaldehyde in PBS for 15 min at room temperature, washed, and permeabilized according to the manufacturer’s instructions. PLA was then performed following the Duolink PLA Fluorescence Protocol (Sigma-Aldrich) using mouse anti-AGO2 (Merck Millipore, 04-642; 1:400) and rabbit anti-GW182/TNRC6A (Bethyl, A302-329A; 1:300) as primary antibodies.

For negative controls, each primary antibody was paired with a species-matched IgG isotype control {rabbit monoclonal antibody (mAb) IgG [Cell Signaling Technology (CST), no. 3900] and mouse mAb IgG2a (CST, no. 61656)}, or a probes-only condition was used. After counterstaining with Phalloidin–Alexa Fluor 488 (AF488; CST, no. 8878) to define cell boundaries and 4′,6-diamidino-2-phenylindole (DAPI) to mark nuclei, coverslips were mounted in Duolink Mounting Media.

Slides were imaged using a ZEISS LSM 880 confocal microscope with Airyscan (64× oil objective). Signal gain and offset were optimized for each experiment relative to negative controls. *Z*-stack images were processed using ZEN 3.4 (blue edition, Zeiss), ImageJ, and CellProfiler, with the latter used for per-cell quantification of uniformly thresholded PLA puncta (cell boundaries defined from F-actin outline and nuclei from DAPI). PLA interaction signal was quantified in ≥8 cells per condition across multiple fields and wells (≥3), and images were digitally adjusted for clarity in figures without altering relative intensities.

### psiCHECK-2 cloning and dual-luciferase reporter assays

Oligonucleotides encoding empty vector (VO), tandem miRNA sites with seed matches (T) or mismatches (NT), or artificial reporters were cloned into the 3′ UTR region of the Renilla luciferase cassette in psiCHECK-2 (Promega, C8021) using Xho I and Not I restriction sites. Tandem miR-99/100 sites (T: 5′-AGCAAGTGTAACGG**TACGGGT**A-3′; NT: 5′-AGCAAGTGTAACGG**TA***ATAAC*A-3′) contained five repeats and were created as previously described (*19*). Artificial reporters included a let-7a construct containing six seed-matched sites (5′-AACTATACAACGT**CTACCTC**A-3′) and a miR-21 construct containing three seed-matched sites (5′-TCAGAAG**ATAAGCT**AGGGGTCA-3′, 5′-TCAGAAG**ATAAGCT**AGGGGTCA-3′, and 5′-TCAGAAG**ATAAGCT**AGTTTAAA-3′). Bold indicates the seed-matched region. Constructs were verified by colony PCR, diagnostic Xho I/Not I digest, and Sanger sequencing, and plasmids were diluted to 100 ng/μl in Tris-EDTA (TE) buffer for transfection.

For luciferase assays, 5000 cells per well were seeded in 96-well plates and transfected 24 hours later with 20 ng of psiCHECK-2 plasmid using Attractene (QIAGEN, 301005) in Opti-MEM (Thermo Fisher Scientific, 31985070). After 24 hours, cells were lysed in 25 μl of Passive Lysis Buffer (Promega, E1960) and subjected to one freeze-thaw cycle. Lysates (20 μl) were transferred to white-walled plates for sequential luminescence measurement on a FLUOstar Omega plate reader (BMG Labtech) with 25 μl of Firefly substrate followed by 25 μl of Renilla substrate. Each condition was assayed in ≥4 technical replicates across ≥3 independent experiments. Renilla activity was normalized to Firefly internal control and expressed relative to VO to calculate miRNA-mediated repression.

### Chimeric miR-eCLIP protocol

Chimeric miR-eCLIP was performed as described previously (*34*). Briefly, 2 × 1.25 million cells from four samples per group (three independent CRISPR clones and one clone repeated) were seeded into 15-cm dishes and grown to ∼80% confluence (∼2 × 10 million cells each) before ultraviolet–cross-linking on ice at 254 nm (400 mJ/cm^2^). Cells were scraped, pelleted at 300*g*, flash frozen in liquid nitrogen, and stored at −80°C. Pellets were lysed in cold eCLIP buffer containing protease and ribonuclease (RNase) inhibitors and then sonicated on a QSonica Q800R (75% amplitude, 30 s on/off for 10 cycles). Lysates were adjusted to fragment RNA with RNase I according to sample RNA integrity number (RIN), clarified by centrifugation, and aliquoted to yield 20 μg of RNA per IP. Immunoprecipitation was carried out overnight at 4°C with AGO2 antibody (Santa Cruz Biotechnology, sc-53521)–coated magnetic beads (M280 Sheep Anti-Mouse IgG Dynabeads; Thermo Fisher Scientific, 11202D). Beads were washed under stringent high-salt and no-salt conditions, sequentially treated with phosphatase (PSP) and polynucleotide kinase (PNK) enzymes to repair RNA ends, and subjected to on-bead chimeric miRNA:mRNA ligation with T4 RNA ligase. Following additional washes, 3′ RNA adapters were ligated on-bead, and RNA was released by proteinase K digestion.

Matched input RNA samples were processed in parallel: column cleanup, PSP/PNK end repair, and 3′ adapter ligation. Both IP and Input RNAs were reverse transcribed using a chimera-specific primer, and the resulting cDNA underwent end repair by nuclease treatment and mild alkaline hydrolysis with bead-based cleanup. Overnight ligation of a single-stranded cDNA adapter was followed by PCR amplification using Illumina i7/i5 index primers, with cycle numbers determined by quantitative PCR (qPCR). Final libraries were purified with AMPure XP beads, quality checked on an Agilent TapeStation, pooled equimolarly, and sequenced single-end (100 bp) on an Illumina platform, targeting ∼50 million reads per IP sample and ∼40 million reads per input sample. Further details on chimeric miR-eCLIP protocol can be obtained upon request.

### Chimeric miR-eCLIP analysis

Initial processing and analysis were performed by Eclipsebio using a proprietary analysis pipeline (v1) developed from several published eCLIP workflows (*34*, *71*, *72*). The original underlying bioinformatic pipelines are publicly available (https://github.com/yeolab/eclip; https://github.com/YeoLab/chim-eCLIP). Briefly, UMIs were pruned with umi_tools (v1.1.1), and 3′ adapters were trimmed with cutadapt (v3.2). Reads of <18 nt after trimming were discarded. Reads were first aligned to a database of repetitive elements and rRNA sequences; nonrepeat reads were then mapped to the human genome (GRCh38/hg38, UCSC) using STAR (v2.7.7a). PCR duplicates were removed with umi_tools. AGO2 eCLIP read clusters were identified with CLIPper (v2.0.1) (*73*), and IP versus input fold enrichment and statistical significance were calculated; clusters meeting predefined thresholds of log_2_ fold enrichment of ≥3 and *P* ≤ 0.001 were designated as AGO2 peaks.

In parallel, nonchimeric reads were used to quantify AGO2-bound miRNA abundance. Reads were adapter-trimmed, UMI deduplicated, and aligned to mature miRNA sequences from miRBase (v22.1) using Bowtie, allowing multimapping to account for homologous family members. Abundance was expressed as reads per million relative to the total AGO2-bound miRNA read count per sample. These data enabled direct comparison of miRNA expression levels between Ctrl, Het, and KO conditions.

Unmapped reads were “reverse mapped” to mature miRNAs from miRBase (v22.1) using Bowtie (v1.2.3). The miRNA sequence was trimmed, and the remainder was aligned to the genome with STAR; PCR duplicates were again removed with umi_tools. miRNA:target clusters were identified with CLIPper and annotated with the responsible miRNA(s). Peaks were annotated using transcript information from GENCODE release 41 (GRCh38.p13) with priority given to protein-coding features (CDS, UTRs, and introns) over noncoding transcripts (exons and introns). AGO2 clusters were normalized to paired input samples, and reproducibility was assessed by IDR analysis. Reproducible chimeric AGO2-miRNA:target peaks were defined as those with ≥3 chimeric reads from the same miRNA seed family overlapping the peak in each replicate.

Each reproducible chimeric peak was, therefore, treated as a unique AGO2-miRNA:target-site interaction with its seed match. After UMI deduplication, each retained chimeric read reflects a unique ligation event captured in the experiment, providing a measure of relative interaction abundance rather than absolute molecular counts (*34*). While the low sensitivity of ligation assays means that many true interactions are not recovered and ligation or sequencing biases may influence capture, UMI deduplication ensures that counts are not inflated by PCR artifacts.

Peak-level data (genomic coordinates, gene, RNA feature, enrichment/chimeric read counts, fold changes, and *P* values) were further analyzed using Microsoft Excel and GraphPad Prism v9.5.1. AGO2-miR-eCLIP input and IP signal tracks (.bigWig files) have been deposited in Gene Expression Omnibus (GEO) under accession number GSE304955, and reproducible AGO2 and chimeric peak data are provided in the Supplementary Materials.

### PEKA analyses

PEKA was applied as described previously (*42*) to reproducible AGO2-miRNA chimeric peaks. Putative cross-link sites (Xn) were partitioned into thresholded sites (tXn; within peaks containing ≥70% of the regional cDNA signal) and reference sites (oXn; outside peaks), with all others discarded. For each set, sequences flanking tXn and oXn (±100 nt) were extracted, scanned for *k*-mer presence, and used to calculate relative occurrence (RtXn). RtXn scores represent fold enrichment of a *k*-mer at each position relative to flanking background (RtXn = 0, no binding; 1, background level; and >1, position-specific enrichment). The PEKA score was defined as the standardized difference between a *k*-mer’s mean RtXn at enriched positions and its mean RtXn from randomly sampled background sites, capturing both the magnitude and positional consistency of enrichment.

For visualization and quantitative comparison across Ctrl, Het, and KO conditions, clustered heatmaps of the top 40 *k*-mers by PEKA score were generated. From these data, additional metrics were derived, including: (i) GC content of the top 40 motifs; (ii) maximal occurrence values, defined as the highest enrichment at centered, upstream, or downstream positions relative to the cross-link site, as indicated by dot positioning on the PEKA heatmap; (iii) mean enrichment per nucleotide position, calculated by averaging enrichment across motifs at each position relative to tXn within each condition; Gaussian smoothing (σ = 3.33) was applied to reduce noise and generate positional enrichment profiles; and (iv) local Shannon entropy, computed in sliding 9 × 9-pixel windows across motif heatmaps, with mean entropy values calculated per motif row. Entropy quantifies variability in positional enrichment, with higher values reflecting smoother, more distributed footprints and lower values reflecting punctate binding profiles. Together, PEKA analysis provides positional resolution of target-motif sequences relative to the AGO2 cross-link site, enabling assessment of how LIMD1 deficiency alters motif positioning around AGO2.

### Canonical seed GC content calculation and matched canonical peak analysis

Canonical seed matches (8-mer, 7-mer-m8, 7-mer-A1, and 6-mer) were identified from chimeric peaks. For each, the corresponding miRNA sequence was retrieved from miRBase (mature.fa), and the 6-nt seed (positions 2 to 7) was extracted. GC content was calculated as the proportion of G/C bases and used to classify interactions as GC poor, intermediate, or GC rich. Matched canonical interactions were here defined as those detected across all conditions with overlapping genomic coordinates (±10 bp). For each matched peak, mean chimeric read counts were obtained per condition, and percentage changes relative to Ctrl were calculated after stratification by GC category.

### mRNA-seq library preparation and analysis

hSAECs (1.25 million cells per 15-cm dish; one dish per CRISPR clone, three clones per group) were cultured for 7 days (media changed three times) to ∼80% confluence (∼10 million cells per dish). After pelleting, total RNA was extracted from frozen pellets using the miRNeasy Mini Kit (QIAGEN), and poly(A) RNA was enriched with Dynabeads Oligo(dT)25 (Thermo Fisher Scientific). RNA integrity was assessed on an Agilent 4200 TapeStation.

For mRNA-sequencing (mRNA-seq) libraries, 50 ng of poly(A) RNA was fragmented in 2× FastAP buffer (Thermo Fisher Scientific), dephosphorylated with FastAP, phosphorylated with T4 PNK (NEB), and ligated at the 3′ end to an RNA adapter with T4 RNA ligase (NEB). After Zymo column cleanup, fragments were reverse transcribed with SuperScript III (Invitrogen), treated with ExoSAP-IT (Affymetrix), and ligated at the cDNA 3′ end to a 5′ Illumina adapter. Libraries were cleaned on Dynabeads MyOne Silane (Thermo Fisher Scientific). Cycle number was determined by qPCR, followed by final amplification with barcoded primers (Q5 polymerase, NEB) and size selection using AMPure XP beads (Beckman). Libraries were quantified on the TapeStation and sequenced on an Illumina NovaSeq.

Reads containing a 10-nt UMI at the 5′ end were processed with umi_tools (v1.0.1), adapter trimmed with cutadapt (v2.7), aligned first to RepBase 18.05 and then to hg38 using STAR (v2.7.6a), and deduplicated by UMI and position with umi_tools. Gene counts were generated against GENCODE v29 annotations, and differential expression was analyzed using DESeq2 (v1.34.0). Bulk mRNA-seq data (csv files containing unnormalized raw gene counts) have been deposited to GEO under the accession number GSE305016.

### Total proteomics

Proteomics experiments were performed using mass spectrometry (MS) as previously reported (*74*, *75*), with minor technical modifications, on six independently plated, lysed, and processed replicates of Ctrl C4, Het C6, and KO C2 hSAECs. Briefly, cells were seeded and grown out in 15-cm dishes for 7 days and pelleted as described as described for chimeric eCLIP. Frozen pellets were lysed in 8 M urea buffer (Sigma-Aldrich) and supplemented with phosphatase inhibitors [10 mM Na_3_VO_4_, 100 mM β-glycerol phosphate, and 25 mM Na_2_H_2_P_2_O_7_ (Sigma-Aldrich)]. Proteins were digested into peptides using trypsin as previously described (*76*, *77*). From each sample, 30 μg of protein was reduced and alkylated sequentially with 10 mM dithiothreitol (1 hour, 25°C, agitation) and 16.6 mM iodoacetamide (30 min, 25°C, dark). Trypsin beads (50% slurry of TLCK-trypsin; Thermo Fisher Scientific) were equilibrated in 20 mM Hepes (pH 8.0), the urea concentration was reduced to 2 M, and 70 μl of beads were added for overnight digestion at 37°C. Beads were removed by centrifugation (2000*g*, 5 min, 5°C). Peptides were desalted using the AssayMAP Bravo platform (Agilent Technologies, peptide clean-up v3.0). Reverse phase S cartridges (Agilent, 5-μl bed volume) were primed with 250 μl of 99.9% acetonitrile (ACN) with 0.1% trifluoroacetic acid (TFA) and equilibrated with 250 μl of 0.1% TFA at a flow rate of 10 μl/min. The samples were loaded at 20 μl/min, followed by an internal cartridge wash with 0.1% TFA at a flow rate of 10 μl/min. Peptides were then eluted with 105 μl of solution (70/30 ACN/H_2_O and 0.1% TFA). Eluted peptide solutions were dried in a SpeedVac vacuum concentrator and stored at −80°C.

Samples were run in the liquid chromatography (LC)–MS system over consecutive days to reduce technical variability. Peptide pellets were reconstituted in 5 μl of 0.1% TFA, after which 2 μl of this solution was further diluted in 18 μl of 0.1% TFA: 2 μl was injected into the LC-MS/MS system. The LC-MS/MS platform consisted of a Dionex Ultimate 3000 RSLC coupled to Q Exactive Plus Orbitrap Mass Spectrometer (Thermo Fisher Scientific) through an EASY-Spray source. Mobile phases for the chromatographic separation of the peptides consisted of solvent A [3% ACN and 0.1% formic acid (FA)] and solvent B (99.9% ACN and 0.1% FA). Peptides were loaded in a μ-precolumn and separated in an analytical column using a gradient running from 3 to 28% B over 90 min. The ultra-performance liquid chromatography system delivered a flow of 10 μl/min (loading) and 250 nl/min (gradient elution). The Q Exactive Plus operated a duty cycle of 2.1 s. Thus, it acquired full scan survey spectra [mass/charge ratio (*m*/*z*) 375 to 1500] with a 70,000 full width at half maximum (FWHM) resolution followed by data-dependent acquisition in which the 15 most intense ions were selected for higher energy collisional dissociation and MS/MS scanning (*m*/*z* 200 to 2000) with a resolution of 17,500 FWHM. A dynamic exclusion period of 30 s was enabled with *m*/*z* window of ±10 parts per million (ppm). MS data collection was carried out using Thermo Fisher Scientific FreeStyle 1.4.

MS raw files were converted into Mascot Generic Format using Mascot Distiller (version 2.8.5) and searched against the UniProt database (UniProt.fasta) restricted to human entries using the Mascot search daemon (version 3.0.0). Allowed mass windows were 10 ppm and 25 millimass units (mmu) for parent and fragment mass to charge values, respectively. Identified peptides were quantified using in-house software Pescal as described previously (*76*). Quantitative data were processed in R (v4.2.2), Excel, and GraphPad Prism v9.5.1. Only peptides identified in all replicates of all groups and mapping uniquely to proteins were included in final analyses (*n* = 2667). The analysis code is available at https://github.com/CutillasLab/protools2.

### Gene-set selection and lung expression and survival analysis for LIMD1-AGO2-miRNA:target signature analyses

To define a LIMD1-AGO2:miRNA-target signature, we hypothesized to be dysregulated in LIMD1-deficient LUAD, we applied the following criteria: in both Het and KO, genes with significantly de-enriched 3′UTR chimeric peaks or within significantly dysregulated targetomes of five miRNAs; increased mRNA expression (*q* < 0.05); increased protein levels (*P* < 0.1); and LIMD1 dose dependency of chimeric read loss, mRNA increase, and protein increase. Five genes met these criteria: *VDAC1*, *HMGB2*, *KIF2A*, *IGFBP3*, and *PPIF*. To avoid bias (i.e., only then further analyzing genes that showed desired results), no other or further selections were performed. For context, four of the five genes have published oncogenic roles in LUAD, and three (*VDAC1*, *HMGB2*, and *KIF2A*) have been directly implicated in miRNA-associated dysregulation in NSCLC (*46*–*52*); this was not used for selection.

Using The Cancer Genome Atlas (TCGA) LUAD mRNA data (normalized log_2_[FPKM-UQ + 1]; *n* = 517 tumor and *n* = 72 normal samples) and CPTAC mRNA and protein datasets (*n* = 99 matched LUAD tumor–NAT paired samples) (*78*), *z*-scores for the five-gene set were computed with the R package GSVA; NAT and tumor expression values were used as the reference. These *z*-scores were then correlated with *LIMD1* mRNA or LIMD1 protein abundance to generate signature Pearson correlations. Survival analyses (Kaplan-Meier, univariate, and multivariate Cox proportional hazards) were performed using TCGA LUAD overall survival and clinical annotation data. The composite score (TCGA LUAD) was calculated as *z*-transformed LIMD1 mRNA expression minus the *z*-transformed target set expression. Patients were dichotomized by upper and lower quartiles (highest versus lowest) for Kaplan-Meier survival analyses. Protein-level survival analysis was not performed because of the absence of public MS-protein survival datasets.

### Evolutionary conservation of miRNAs, target sites, and thermodynamic stability of miRNA:target duplexes

The ages of miRNA loci and families were obtained from miRGeneDB 2.1 (*28*) and species divergence dates were taken as the median values reported in TimeTree 5 (*79*). LIMD1-dependent canonical interactions were defined as those showing reduced enrichment (*P* < 0.3) in chimeric eCLIP data in KO versus Ctrl. Logistic regression models were constructed as [retained/lost in *LIMD1* KO] ∼ expression_level + conservation + interaction. Expression level was defined from average miRNA abundance in Ctrl cells and classified into three groups: highly expressed (top 10%), lowly expressed (bottom 10%), and mid-level (remaining 80%). Mature miRNA sequences were obtained from miRBase v22 (*80*). Canonical target predictions were generated with seedVicious v1.1 (*81*), which was also used to compute predicted miRNA:target duplex hybridization energies using the ViennaRNA package (*82*). Conservation of AGO2-miRNA:target sites was measured as phyloP scores (*55*) at the first position (3′) of the target seed match (6-mer), derived from the vertebrate 100-way alignment (hg38; https://hgdownload.soe.ucsc.edu/goldenPath/hg38/phyloP100way/). GC seed content of canonical interactions was calculated as described above.

### GO:MF analysis

Canonical LIMD1-dependent sites (reduced enrichment in KO versus Ctrl; *P* < 0.3) with negative phyloP scores were subjected to Gene Ontology molecular function (GO:MF) enrichment analysis using GOrilla (http://cbl-gorilla.cs.technion.ac.il), with all genes identified in mRNA-seq used as the background set. Enriched GO:MF terms were retained at false discovery rate (FDR) *q* < 0.001. To reduce redundancy, significant terms were clustered with REViGO (http://revigo.irb.hr). Final GO categories were grouped manually into DNA-binding/transcription-related and small-molecule/ion-binding functions. Genes encoding C_2_H_2_-ZNF proteins were assigned according to HUGO Gene Nomenclature Committee gene group annotations.

### Statistical analyses

Statistical analyses were performed after assessing data normality and selecting tests appropriate to experimental design and data structure. Multiple-comparison corrections were applied where required. Statistical values are reported in the figures and legends. All analyses were conducted in GraphPad Prism v9.5.1 or Python, except for miR-eCLIP differential enrichment and mRNA-seq differential expression, which were analyzed with DESeq2 as described above.

### Use of AI-assisted tools

Google Gemini 3 (thinking mode) was used in limited instances to assist with Python code generation and troubleshooting for selected analyses. The resulting author-validated code was then used to generate analysis outputs and corresponding figures. No AI tools were used to generate figure images directly.

## AI-assisted manuscript preparation disclosure

OpenAI’s ChatGPT 5 Pro (version as of March 2026) was used to refine the clarity, tone, and concision of author-written manuscript text and figure captions. A representative prompt used for this purpose was: “Please help refine the clarity, cogency, and conciseness of the following scientific text without altering its meaning or scientific content. Maintain a formal academic tone appropriate for a high-impact journal.” AI tools were not used to generate scientific conclusions, interpret data, manipulate figures, or create images or multimedia. All AI-assisted outputs were critically reviewed by the authors, who take full responsibility for the accuracy and integrity of the manuscript.

## Supplementary Materials

### The PDF file includes:

Figs. S1 to S10 Tables S1 to S5 Legends for supplementary Excel files S1 to S5

### Other Supplementary Material for this manuscript includes the following:

Supplementary Excel files S1 to S5
