# Supplementary material for: The AGO2 adaptor LIMD1 expands the functional and evolutionary reach of microRNA targeting
Source: Sci Adv. 2026 Jul 23;12(30):eaed6875. doi: 10.1126/sciadv.aed6875 (PMC13394485; doi:10.1126/sciadv.aed6875)
Supplement: Supplementary file 1 — Figs. S1 to S10 Tables S1 to S5 Legends for supplementary Excel files S1 to S5 [file sciadv.aed6875_sm.pdf]

Science Advances
AAAS

Supplementary Materials for

The AGO2 adaptor LIMD1 expands the functional and evolutionary reach of microRNA targeting

Alex F. F. Crozier *et al.*

Corresponding author: Kunal M. Shah, k.shah@qmul.ac.uk; Sam Griffiths-Jones, sam.griffiths-jones@manchester.ac.uk; Antonio Marco, amarco@essex.ac.uk; Tyson V. Sharp, t.sharp@qmul.ac.uk

Sci. Adv. **12**, eaed6875 (2026)
DOI: 10.1126/sciadv.aed6875

The PDF file includes:

Figs. S1 to S10
Tables S1 to S5
Legends for supplementary Excel files S1 to S5

Other Supplementary Material for this manuscript includes the following:

Supplementary Excel files S1 to S5

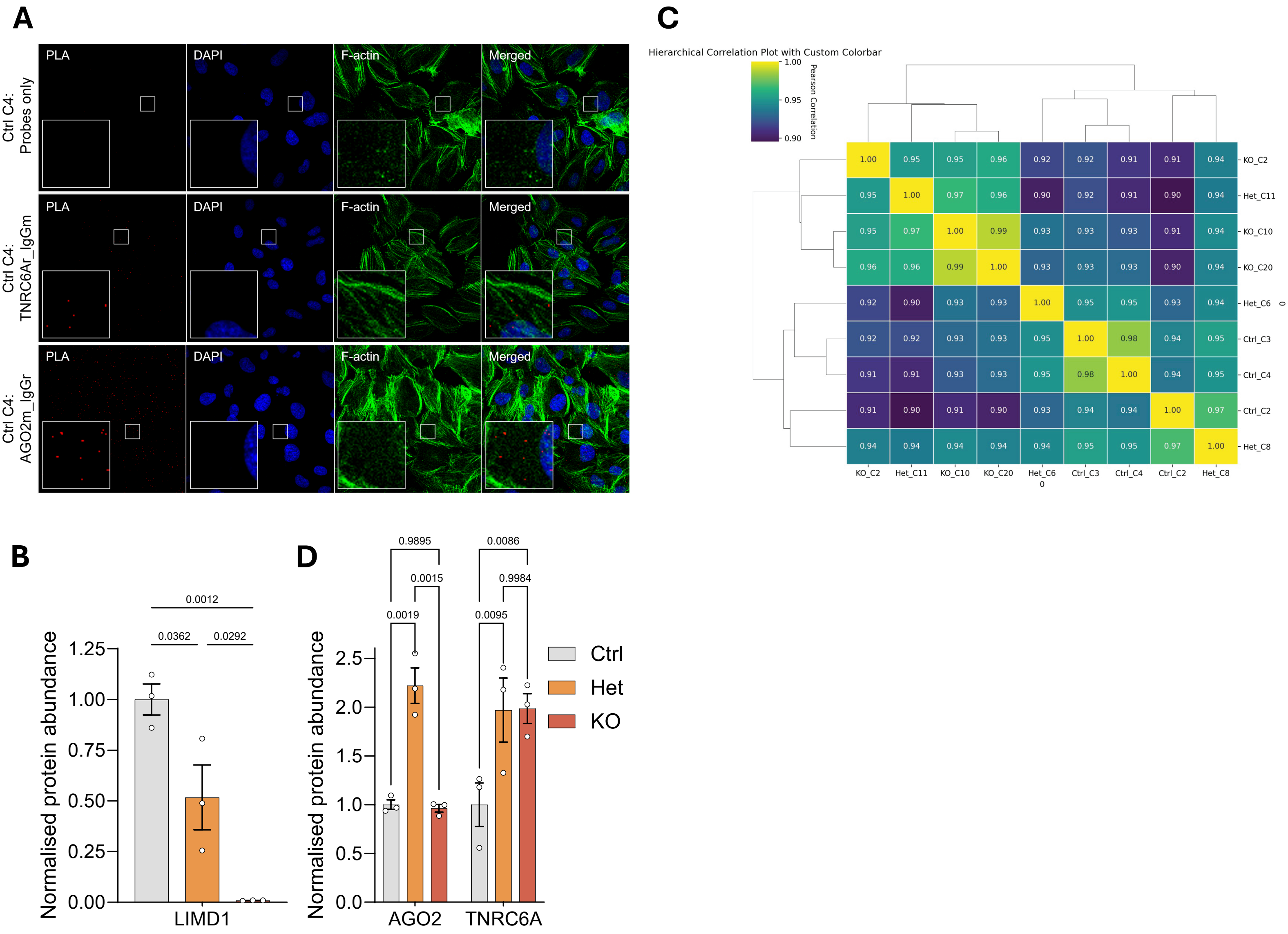

Figure S1. LIMD1 potentiates AGO2–TNRC6A interactions.

(A) Representative images of proximity ligation assay (PLA) controls for AGO2:TNRC6A, shown with PLA signal, DAPI, and F-actin channels. Probes only control contains no primary antibody. IgGm and IgGr denote mouse and rabbit immunoglobulin negative controls, respectively, used to assess background signal as quantified in Figure 1E. **(B)** Immunoblot quantification of LIMD1 protein in Ctrl, Het, and KO CRISPR-hSAECs (as presented in Figure 1F) normalised to vinculin and Ctrl mean (mean of three CRISPR-biological replicates per genotype ± SEM, n=3). LIMD1 was tested by one-way ANOVA. **(C)** Hierarchical clustering of hSAEC RNA-seq sample transcriptomes. A heatmap displays Pearson correlation coefficients between normalized mRNA sequencing read counts of individual hSAEC-CRISPR clone samples. Unsupervised hierarchical clustering was applied to both rows and columns, revealing strong correlation within samples of the same genotype. **(D)** Immunoblot quantification of AGO2 and TNRC6A in Ctrl, Het, and KO CRISPR-hSAECs (presented in Figure 1F) normalised to vinculin and Ctrl mean (mean of three CRISPR-biological replicates per genotype ± SEM, n=3). AGO2 and TNRC6A tested by two-way ANOVA with multiple-comparisons correction.

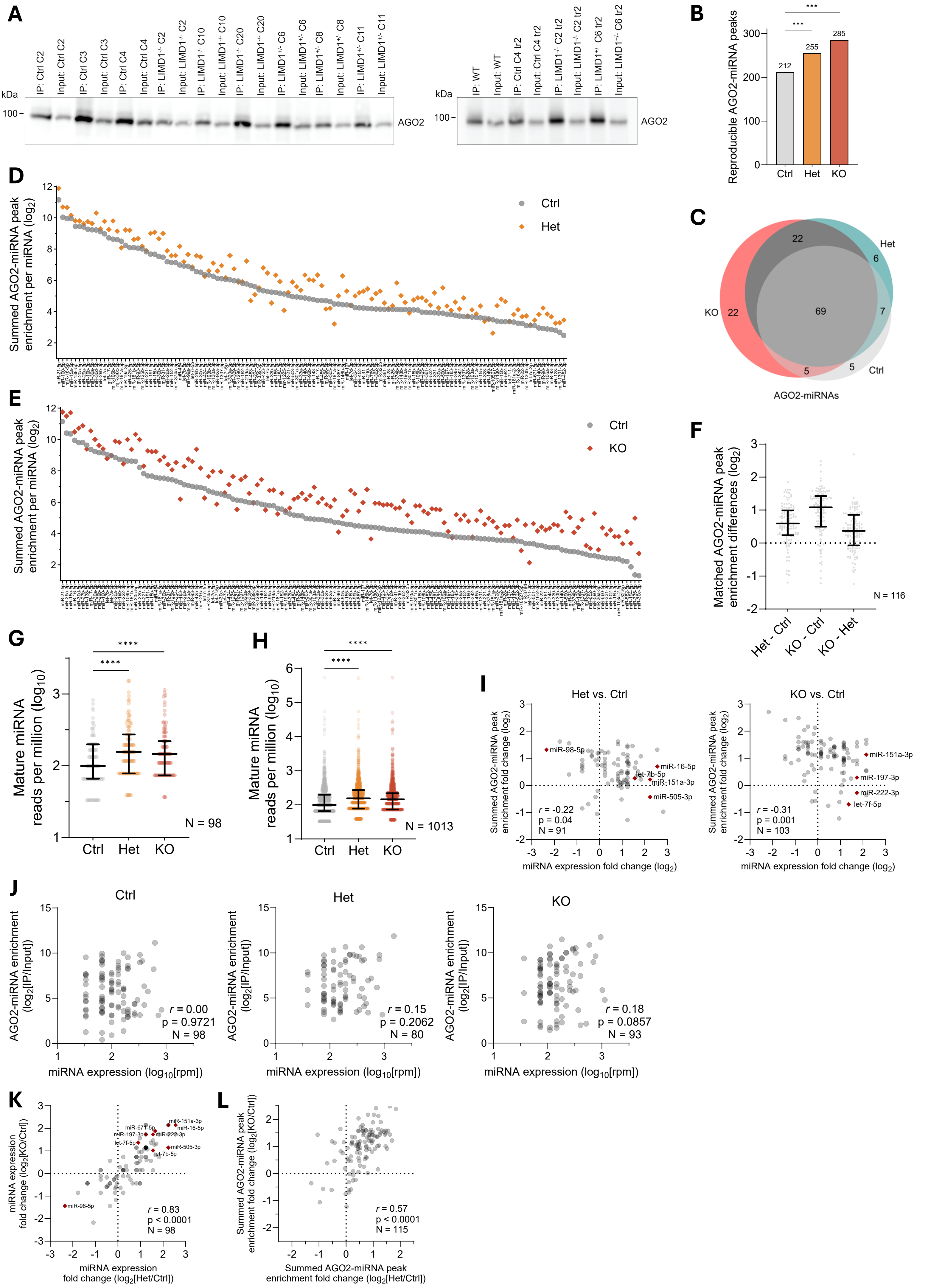
A
kDa
100
IP: Ctrl C2
Input: Ctrl C2
IP: Ctrl C3
Input: Ctrl C3
IP: Ctrl C4
Input: Ctrl C4
IP: LIMD1-/- C2
Input: LIMD1-/- C2
IP: LIMD1-/- C10
Input: LIMD1-/- C10
IP: LIMD1-/- C20
Input: LIMD1-/- C20
IP: LIMD1+/- C6
Input: LIMD1+/- C6
IP: LIMD1+/- C8
Input: LIMD1+/- C8
IP: LIMD1+/- C11
Input: LIMD1+/- C11
AGO2
IP: WT
Input: WT
IP: Ctrl C4 tr2
Input: Ctrl C4 tr2
IP: LIMD1-/- C2 tr2
Input: LIMD1-/- C2 tr2
IP: LIMD1+/- C6 tr2
Input: LIMD1+/- C6 tr2
AGO2
B
Reproducible AGO2-miRNA peaks
***
***
212
255
285
Ctrl
Het
KO
C
22
Het
6
KO
22
69
7
5
5
Ctrl
AGO2-miRNAs
D
Summed AGO2-miRNA peak enrichment per miRNA (log2)
Ctrl
Het
E
Summed AGO2-miRNA peak enrichment per miRNA (log2)
Ctrl
KO
F
Matched AGO2-miRNA peak enrichment differences (log2)
N = 116
Het - Ctrl
KO - Ctrl
KO - Het
G
Mature miRNA reads per million (log10)
****
****
N = 98
Ctrl
Het
KO
H
Mature miRNA reads per million (log10)
****
****
N = 1013
Ctrl
Het
KO
I
Het vs. Ctrl
Summed AGO2-miRNA peak enrichment fold change (log2)
miR-98-5p
miR-16-5p
let-7b-5p
miR-151a-3p
miR-505-3p
r = -0.22
p = 0.04
N = 91
miRNA expression fold change (log2)
KO vs. Ctrl
Summed AGO2-miRNA peak enrichment fold change (log2)
miR-151a-3p
miR-197-3p
miR-222-3p
let-7f-5p
r = -0.31
p = 0.001
N = 103
miRNA expression fold change (log2)
J
Ctrl
AGO2-miRNA enrichment (log2[IP/Input])
r = 0.00
p = 0.9721
N = 98
miRNA expression (log10[rpm])
Het
AGO2-miRNA enrichment (log2[IP/Input])
r = 0.15
p = 0.2062
N = 80
miRNA expression (log10[rpm])
KO
AGO2-miRNA enrichment (log2[IP/Input])
r = 0.18
p = 0.0857
N = 93
miRNA expression (log10[rpm])
K
miRNA expression fold change (log2[KO/Ctrl])
miR-151a-3p
miR-671-5p
miR-16-5p
miR-197-3p
miR-222-3p
let-7f-5p
miR-505-3p
let-7b-5p
miR-98-5p
r = 0.83
p < 0.0001
N = 98
miRNA expression fold change (log2[Het/Ctrl])
L
Summed AGO2-miRNA peak enrichment fold change (log2[KO/Ctrl])
r = 0.57
p < 0.0001
N = 115
Summed AGO2-miRNA peak enrichment fold change (log2[Het/Ctrl])

Figure S2. LIMD1 deficiency increases AGO2–miRNA interactions independently of miRNA expression.

(A) AGO2-IP enrichment over input across chimeric-eCLIP replicates. **(B)** Dose-dependent increase in reproducible AGO2–miRNA peaks in LIMD1-deficient cells (pairwise proportion test, Benjamini–Hochberg correction; ***$P<0.001$). **(C)** Venn diagram of AGO2-associated miRNAs across Ctrl, Het, and KO. **(D, E)** Increased summed peak enrichment per AGO2–miRNA in Het and KO versus Ctrl. **(F)** Enrichment differences for 116 peaks shared by Ctrl, Het, and KO, demonstrating a LIMD1-dosage associated increase in AGO2–miRNA binding. **(G, H)** Global increase in mature miRNA expression (reads per million, $\log_{10}$) for AGO2-associated miRNAs **(G)** and all mature miRNAs **(H)**. **(I)** Moderate negative correlation between AGO2–miRNA enrichment and mature miRNA expression changes (Spearman), indicating expression alone does not drive enrichment. **(J)** Within groups, AGO2–miRNA enrichment correlates poorly or not at all with miRNA expression (Spearman), indicating AGO2–miRNA loading determinants beyond miRNA expression levels. **(K, L)** Positive correlation of changes in miRNA expression **(K)** and summed AGO2–miRNA enrichment **(L)** in Het and KO versus Ctrl (Spearman), consistent with LIMD1-specific effects.

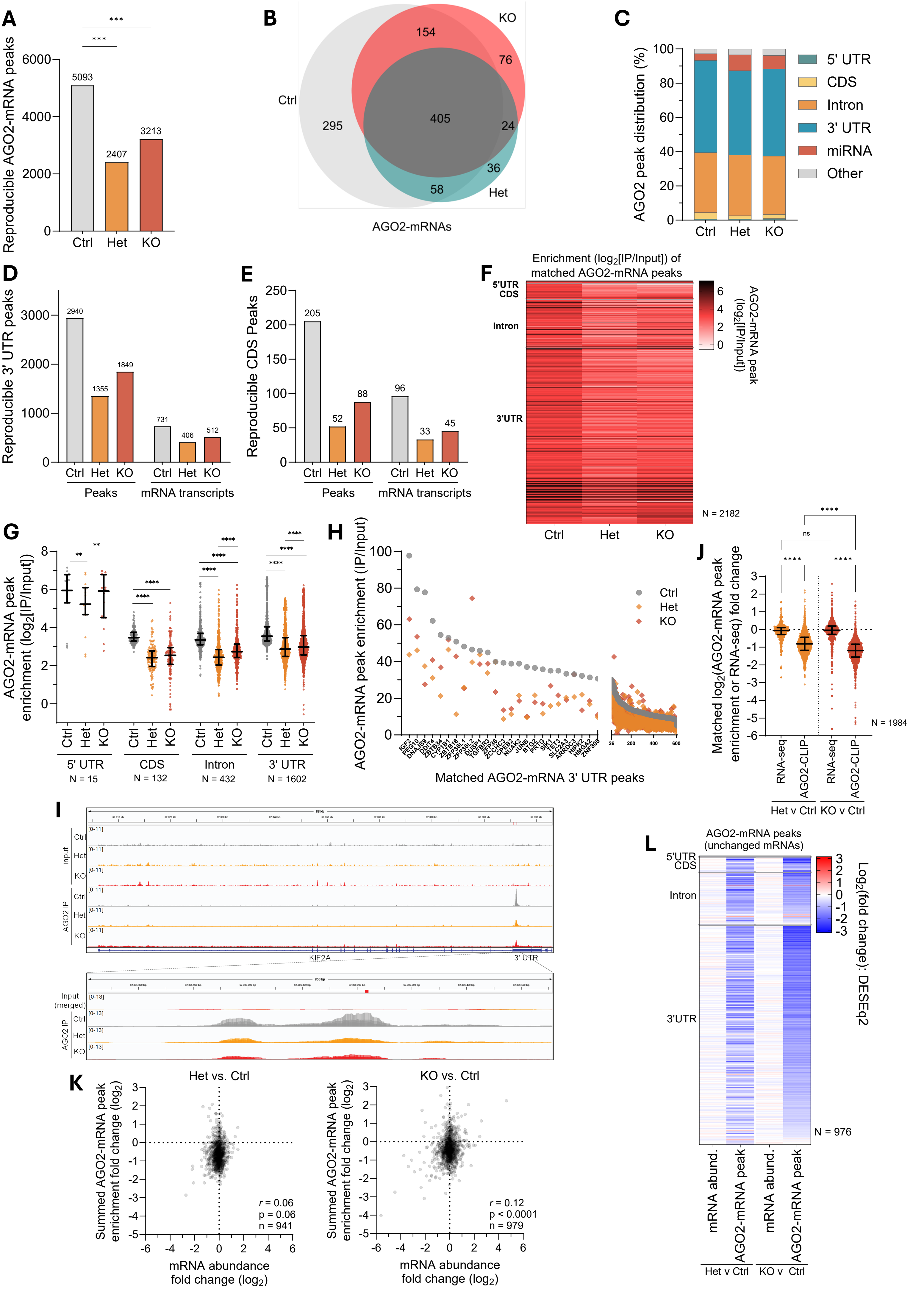
A
Reproducible AGO2-mRNA peaks
5093
2407
3213
Ctrl
Het
KO
***
***
B
KO
Ctrl
Het
154
76
295
405
24
36
58
AGO2-mRNAs
C
AGO2 peak distribution (%)
5' UTR
CDS
Intron
3' UTR
miRNA
Other
D
Reproducible 3' UTR peaks
2940
1355
1849
731
406
512
Peaks
mRNA transcripts
E
Reproducible CDS Peaks
205
52
88
96
33
45
F
Enrichment (log2[IP/Input]) of matched AGO2-mRNA peaks
5'UTR
CDS
Intron
3'UTR
AGO2-mRNA peak (log2[IP/Input])
N = 2182
G
AGO2-mRNA peak enrichment (log2[IP/Input])
5' UTR
N = 15
CDS
N = 132
Intron
N = 432
3' UTR
N = 1602
**
****
H
AGO2-mRNA peak enrichment (IP/Input)
Matched AGO2-mRNA 3' UTR peaks
IGF2
PEG10
DNAJB9
DDIT4
ZBTB34
CYP1B1
ZBTB16
ZFP36L1
ZFP36L2
DUSP1
TGFBR3
ZFP36
ZCCHC3
CREB1
NUAK1
JUNB
BTG2
PRTG
SIK1
TET3
SLC2A3
ARRDC3
HIPK2
HMGA2
ZNF805
J
Matched log2(AGO2-mRNA peak enrichment or RNA-seq) fold change
RNA-seq
AGO2-CLIP
Het v Ctrl
KO v Ctrl
ns
N = 1984
I
88 kb
input
AGO2 IP
[0-11]
KIF2A
3' UTR
850 bp
Input (merged)
[0-13]
K
Het vs. Ctrl
KO vs. Ctrl
Summed AGO2-mRNA peak enrichment fold change (log2)
mRNA abundance fold change (log2)
r = 0.06
p = 0.06
n = 941
r = 0.12
p < 0.0001
n = 979
L
AGO2-mRNA peaks (unchanged mRNAs)
Log2(fold change): DESeq2
N = 976
mRNA abund.
AGO2-mRNA peak
Het v Ctrl
KO v Ctrl

Figure S3. LIMD1 deficiency reduces AGO2–mRNA interactions.

(A) Fewer AGO2–mRNA peaks indicate reduced distinct AGO2–mRNA interactions in LIMD1-deficient cells (pairwise proportion test, Benjamini–Hochberg correction; ***P*<0.001). **(B)** Venn diagram of AGO2-associated mRNAs in Ctrl, Het, and KO, showing reduced repertoire in LIMD1-deficient cells. **(D, E)** Reproducible AGO2–mRNA peaks and distinct transcripts mapping to **(D)** 3′UTRs and **(E)** CDS's. **(F, G)** Enrichment (log_2FC AGO2 IP/Input) for 2,182 matched AGO2–mRNA peaks, shown as a heatmap **(F)** and boxplot **(G)** stratified by genomic region. **(H)** Per-peak enrichment of 606 matched 3′UTR peaks; for transcripts with multiple peaks, only the most enriched is plotted. (**I**) IGV browser tracks of input and AGO2 IP reads across the full KIF2A transcript (top) and the 3′UTR region containing reproducible AGO2–mRNA peaks (bottom). Replicates are overlaid within each genotype for clarity; in the lower panel, input samples are displayed as a single merged track to simplify comparison across the peak-containing region. **(J)** AGO2–mRNA peaks show greater de-enrichment than transcript-level changes, indicating loss of binding is not driven by reduced mRNA abundance (Wilcoxon matched-pairs signed-rank test; ****P*<0.0001). **(K)** Low correlation (Spearman) between mRNA abundance changes and summed AGO2–mRNA peak enrichment confirms major changes not driven by transcript levels. **(L)** Heatmap of log_2 fold changes for mRNAs with stable abundance (0.8–1.2-fold, q>0.9), grouped by genomic feature and ordered by de-enrichment in KO, showing widespread peak loss among stable mRNA transcripts.

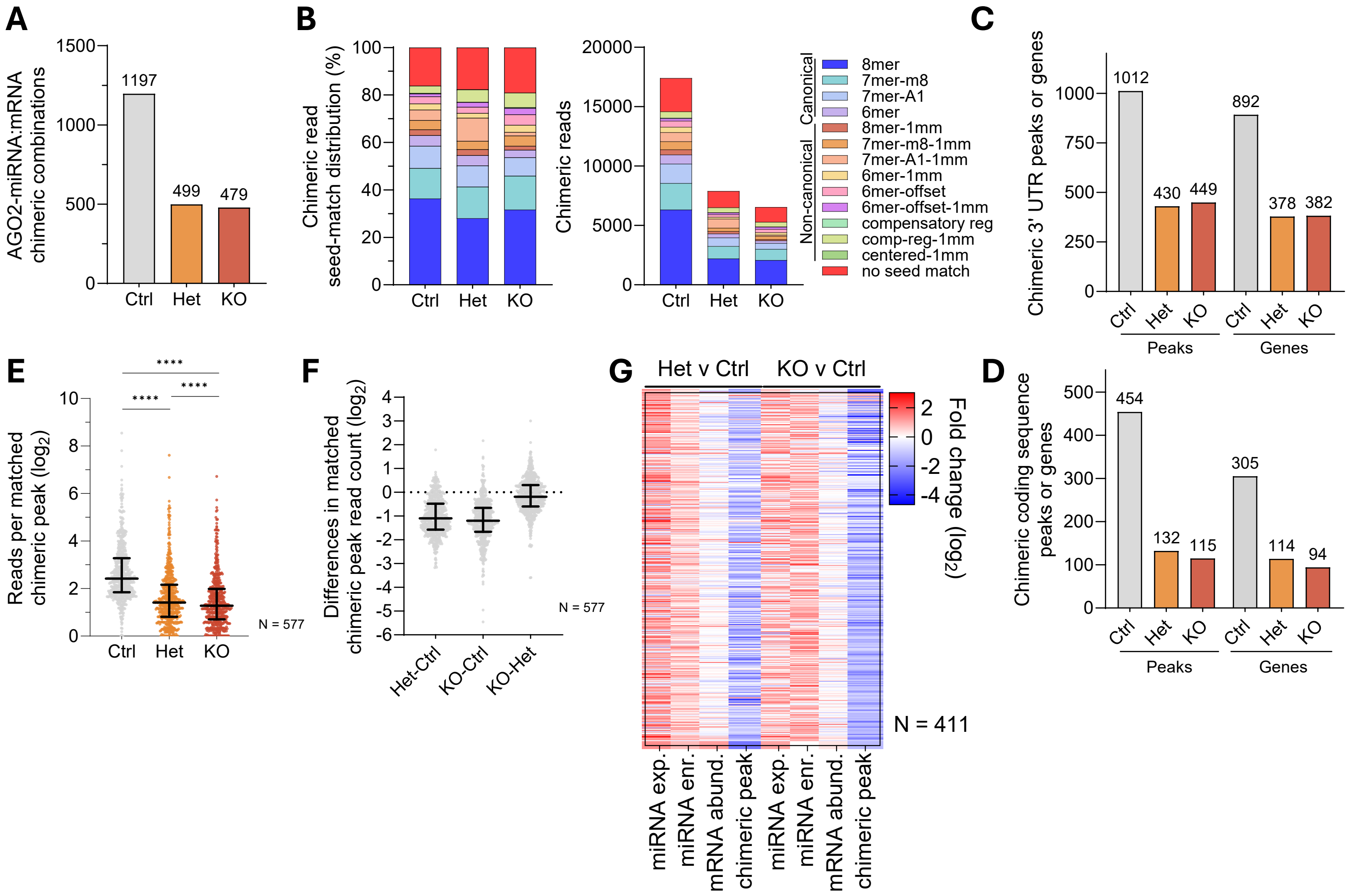

Figure S4. LIMD1 deficiency constrains AGO2–miRNA targeting.

(A) Fewer unique AGO2–miRNA:mRNA combinations in LIMD1-deficient cells. **(B)** Distribution of chimeric read counts by exact seed-match type (TargetScan definition) shows broad reductions across classes in LIMD1-deficient cells. **(C, D)** Fewer reproducible chimeric peaks and unique target transcripts mapping to **(C)** 3′UTR and **(D)** CDS target-sites. **(E, F)** Normalised reads **(E)** and differences **(F)** per matched chimeric peak (≥3 reads in any group, pseudo + 0.01 added to peaks with 0 reads to avoid log0 values; Wilcoxon matched pairs signed-rank test; ****P<0.0001). **(G)** Heatmap of $\log_2$ fold changes in miRNA expression (miRNA exp.), AGO2–miRNA enrichment (miRNA enr.), mRNA abundance (mRNA abund.), and chimeric reads for matched chimeric peaks with complete data in Het and KO versus Ctrl.

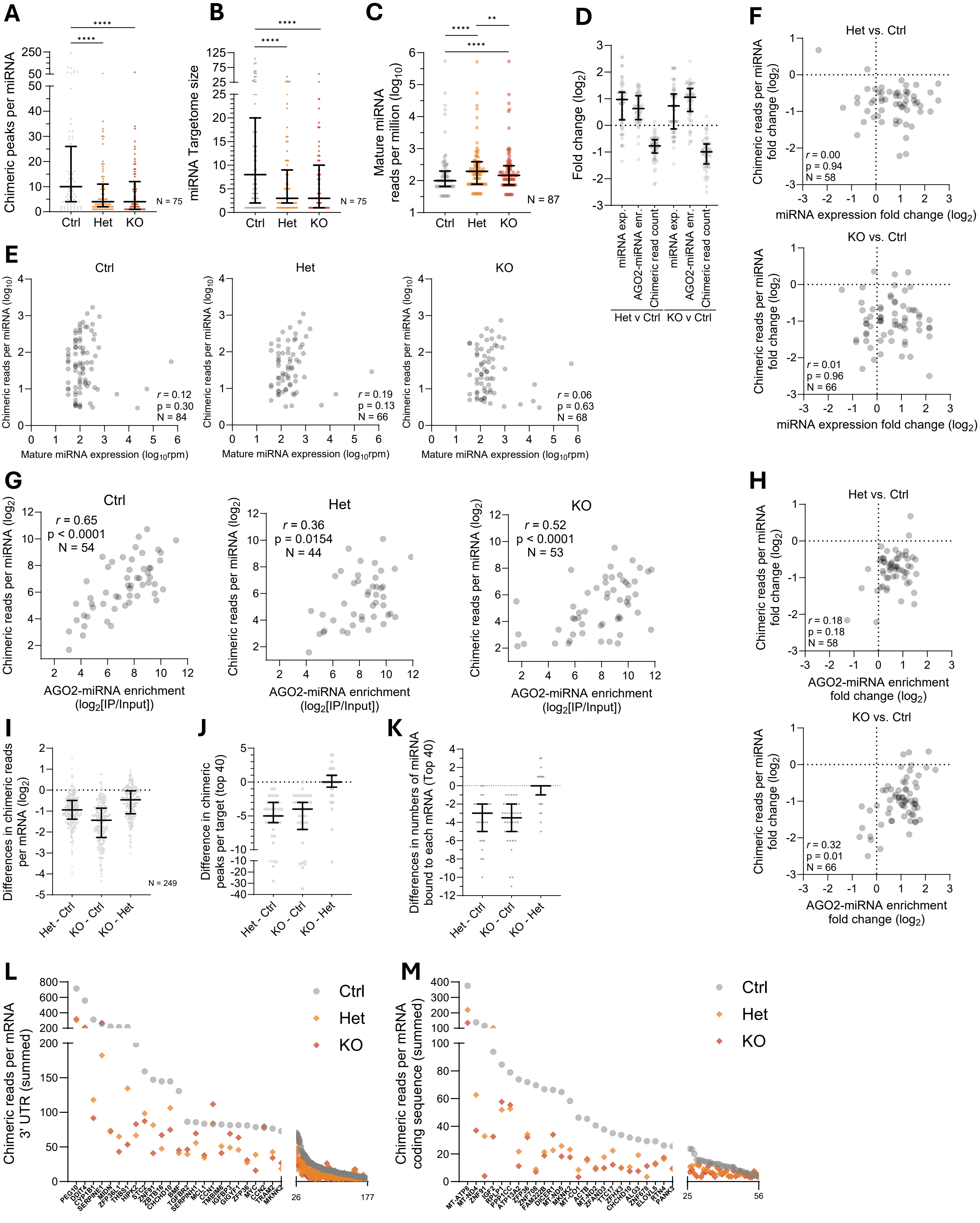
A
Chimeric peaks per miRNA
Ctrl
Het
KO
N = 75
****
B
miRNA Targetome size
N = 75
C
Mature miRNA reads per million (log10)
N = 87
**
D
Fold change (log2)
miRNA exp.
AGO2-miRNA enr.
Chimeric read count
Het v Ctrl
KO v Ctrl
E
Chimeric reads per miRNA (log10)
Mature miRNA expression (log10rpm)
r = 0.12
p = 0.30
N = 84
r = 0.19
p = 0.13
N = 66
r = 0.06
p = 0.63
N = 68
F
Het vs. Ctrl
KO vs. Ctrl
Chimeric reads per miRNA fold change (log2)
miRNA expression fold change (log2)
r = 0.00
p = 0.94
N = 58
r = 0.01
p = 0.96
N = 66
G
Chimeric reads per miRNA (log2)
AGO2-miRNA enrichment (log2[IP/Input])
r = 0.65
p < 0.0001
N = 54
r = 0.36
p = 0.0154
N = 44
r = 0.52
p < 0.0001
N = 53
H
AGO2-miRNA enrichment fold change (log2)
r = 0.18
p = 0.18
N = 58
r = 0.32
p = 0.01
N = 66
I
Differences in chimeric reads per miRNA (log2)
N = 249
Het - Ctrl
KO - Ctrl
KO - Het
J
Difference in chimeric peaks per target (top 40)
K
Differences in numbers of miRNA bound to each mRNA (Top 40)
L
Chimeric reads per mRNA 3' UTR (summed)
PEG10
DDIT4
CYP1B1
SERPINE1
MIDN
ZFP36L1
THBS1
HIPK2
STC2
ZNF91
ZBTB16
CHCHD10
BMF
TGFBR2
SERPINH1
MCL1
CCN1
TMBIM6
IGFBP3
GIGYF1
ZFP36
MYC
CCN2
TRAM2
MKNK2
26
177
M
Chimeric reads per mRNA coding sequence (summed)
MT-ATP8
MT-ND4
ZNF91
IGF2
RPLP1
PPP1CC
ATP1A2
ZFP36
ZNF708
FAM222B
DICER1
MT-ND5
MKNK2
MT-CO1
ACTB
MT-ND2
ZFAND3
TTC17
ZFHX3
CHCHD10
ALG3
ZNF678
ELOVL5
RTN4
PANK3
25
56

Figure S5. LIMD1 deficiency reduces breadth and depth of targeting by each AGO2–miRNA.

(A, B) Chimeric peaks **(A)** and target-transcripts **(B)** per AGO2–miRNA, with paired differences (Wilcoxon matched-pairs signed-rank test; ****P<0.0001). **(C)** General trend of increased mature miRNA expression per miRNA for miRNAs detected by chimeric-eCLIP (Wilcoxon test; **P<0.01, ****P<0.0001). **(D)** Fold change ($\log_2$) in miRNA expression, AGO2–miRNA enrichment, and chimeric reads, showing widespread loss of target binding despite increased expression and AGO2 loading. **(E)** Lack of correlation (Spearman) between miRNA expression ($\log_{10}$ RPM) and chimeric reads, indicating expression does not primarily drive targeting. **(F–H)** Correlation analyses of expression, AGO2 loading, and targeting. **(F)** No correlation between changes in expression and chimeric reads per miRNA in Het or KO versus Ctrl. **(G)** Positive correlation between AGO2 enrichment and chimeric reads, linking loading to targeting. **(H)** Positive moderate correlation between AGO2 enrichment changes and chimeric reads, showing increased loading may partially offset targeting loss, though most miRNAs still show reduced reads. Overall, despite increased miRNA expression and AGO2 loading, chimeric reads declined. **(I–K)** Differences in total chimeric reads, peaks, and number of bound-miRNAs per target transcript. **(L, M)** Reduced chimeric reads per transcript at the **(L)** 3′UTR and **(M)** CDS, showing systematically less AGO2–miRNA binding across both regions of ~all target-mRNAs in LIMD1-deficient cells.

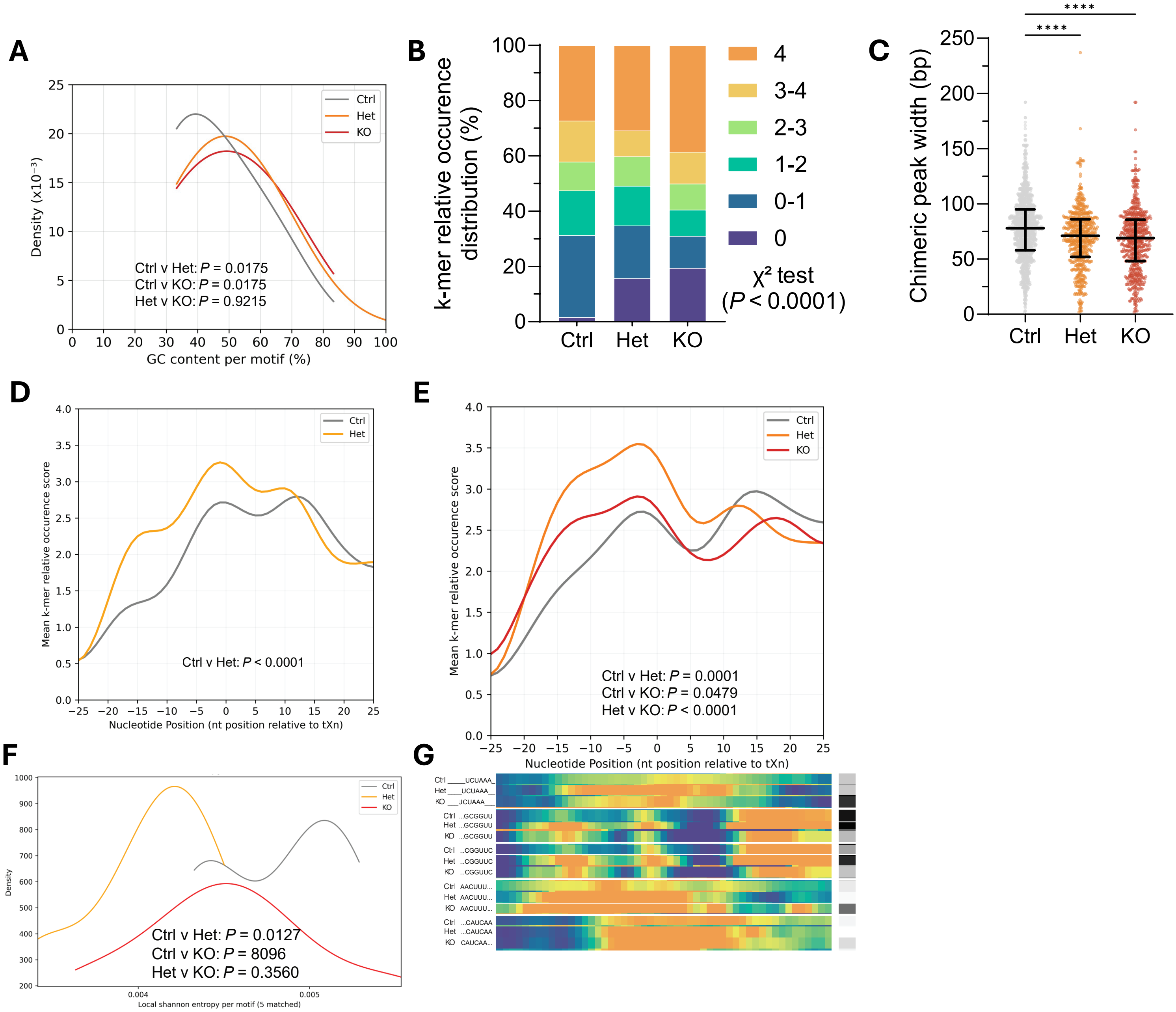

Figure S6. LIMD1 shapes AGO2–miRNA targeting landscapes by governing target motif identity and positional footprint

(A) Seed GC content of canonical interactions, showing a shift toward GC-rich motifs among interactions retained in LIMD1-deficient cells (kernel density, Gaussian σ = 3.33; Kruskal–Wallis with multiplicity correction). **(B)** Distribution of RtXn values (0 = no binding, 1 = background, 4 = strong enrichment), with more zero-occurrence motifs and fewer with intermediate enrichment (1–<4) in LIMD1-deficient cells (χ^2 test). **(C)** Width of reproducible chimeric peaks, in base pairs, in Ctrl, Het, and KO cells. Statistical significance was assessed by Kruskal–Wallis test with Dunn's multiple-comparisons correction; ****, $P <$ 0.0001. **(D, E)** Motifs shared between Ctrl and Het (n = 9) **(D)** or Ctrl, Het, and KO (n = 5) **(E)** show an upstream (5′) shift in binding relative to tXn in LIMD1-deficient cells, even for identical motifs (Wilcoxon test). **(F, G)** Shannon entropy per motif for the five motifs common to all groups indicates that LIMD1 deficiency produces a sharper, punctate binding footprint even among identical target-motifs (corrected matched one-way ANOVA).

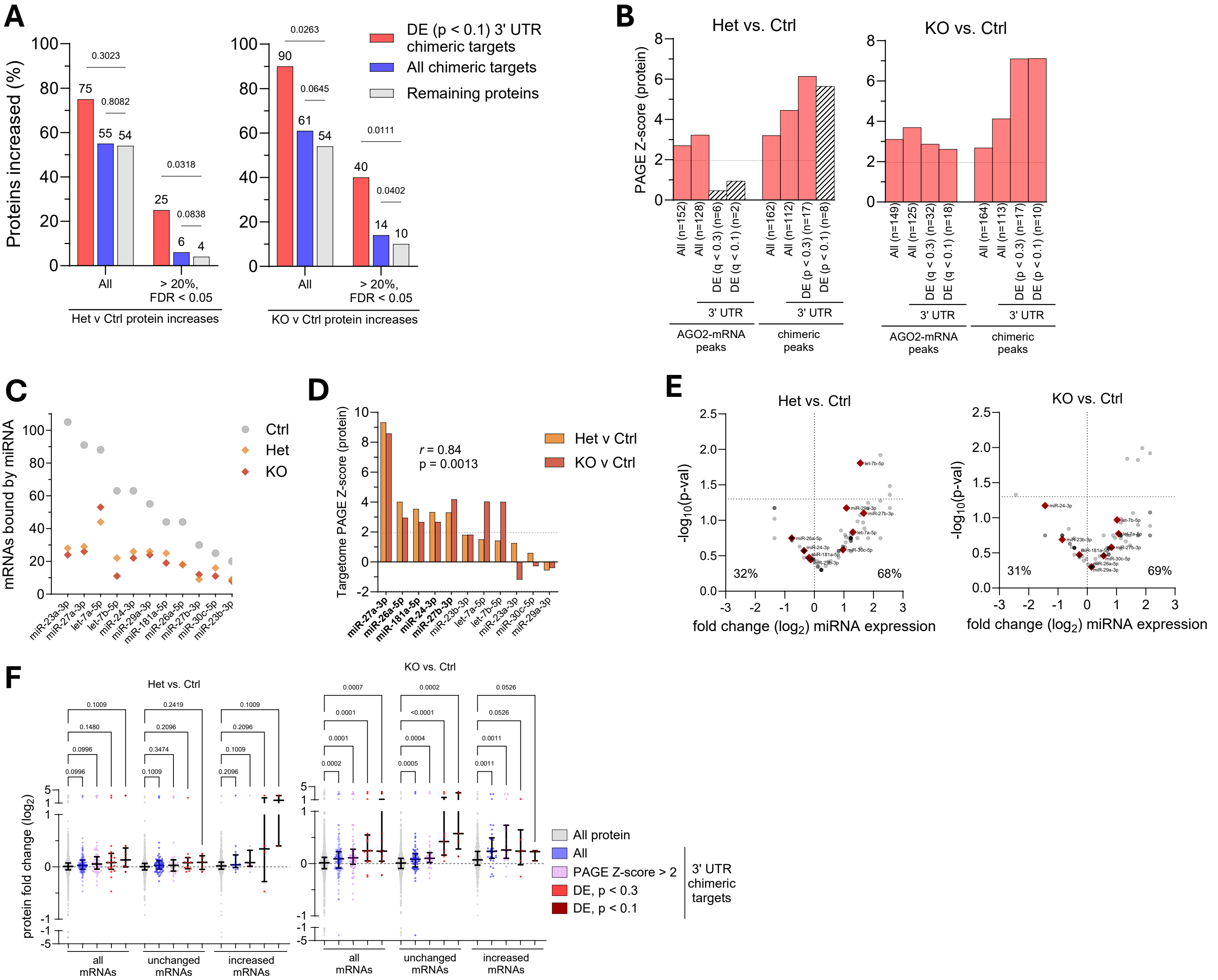

Figure S7. LIMD1 deficiency de-represses miRNA targets in hSAECs.

(A) Enrichment of all chimeric targets and top de-enriched (P<0.1) 3′UTR chimeric targets among increased proteins ($log_2FC>0$) and proteins increased >20% with FDR<0.05 (Fisher's exact test). **(B)** PAGE Z-scores showing significant net protein increases for AGO2–miRNA targets in Het and KO versus Ctrl. Higher Z-scores were observed for chimeric versus AGO2–mRNA targets, 3′UTR versus all peaks, and top de-enriched 3′UTR peaks versus all 3′UTRs; Z>1.96 (dotted line) indicates significance ($P<0.05$). Dashed bars indicate insufficient protein numbers for PAGE analysis. **(C)** Fewer experimentally identified target mRNAs per miRNA in Het or KO versus Ctrl for miRNAs included in PAGE analysis. **(D)** Strong correlation of protein changes between Ctrl versus Het and Ctrl versus KO (Pearson r=0.84, P=0.0013), with consistently increased targetomes (PAGE Z>1.96), indicating LIMD1-specific effects. **(E)** Expression changes of miRNAs with derepressed targetomes show several with increased abundance, confirming derepression is not due to reduced miRNA levels. **(F)** Protein fold changes (log_2) for 3′UTR chimeric targets (blue), targets of miRNAs with PAGE Z>2 (purple), and de-enriched chimeric targets (P<0.1, dark red), across all, unchanged (0.8–1.2-fold), and increased (>1.2-fold) mRNAs, showing elevated protein levels even without mRNA changes (Kruskal–Wallis with correction).

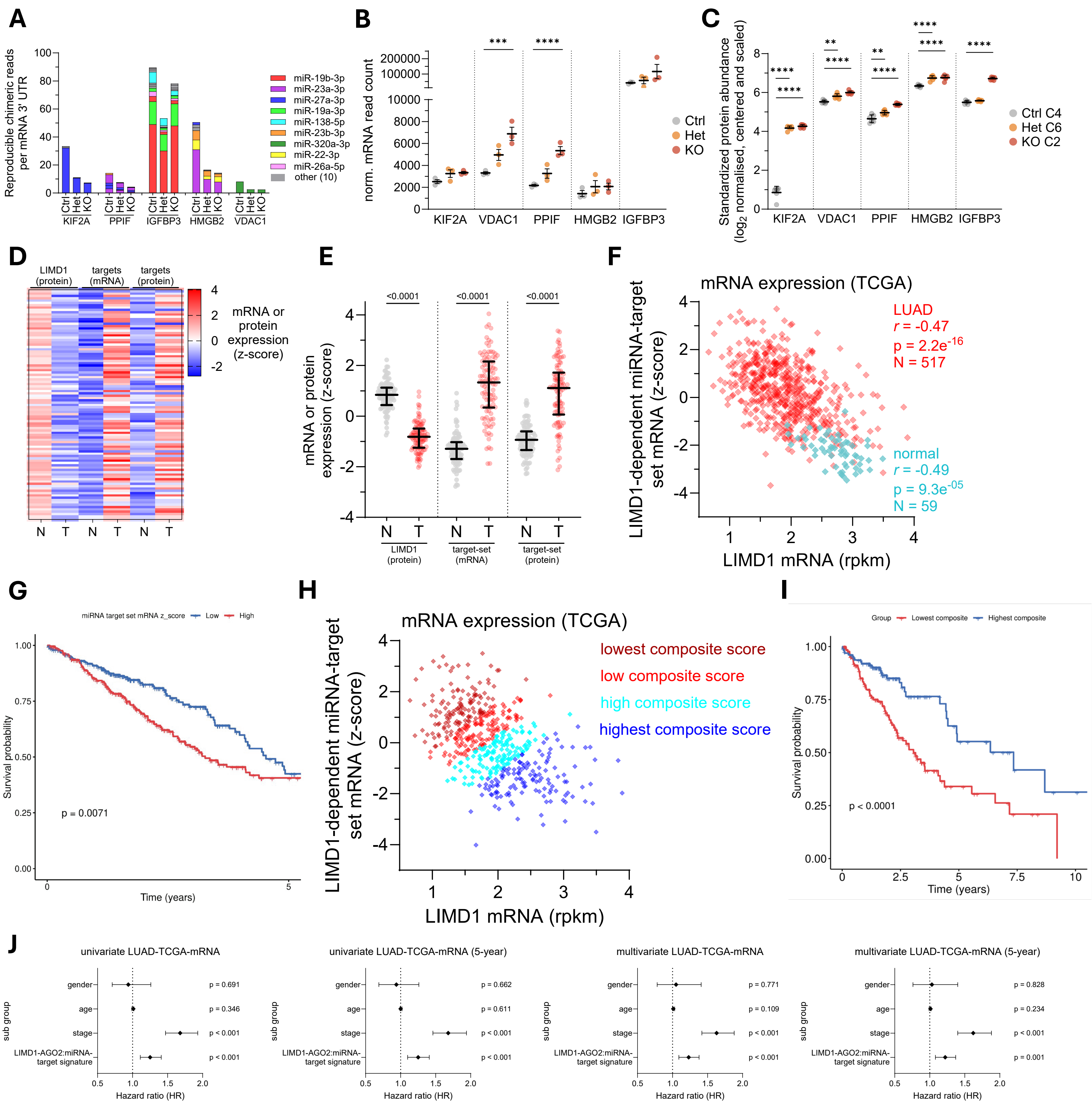
A
Reproducible chimeric reads per mRNA 3' UTR
miR-19b-3p
miR-23a-3p
miR-27a-3p
miR-19a-3p
miR-138-5p
miR-23b-3p
miR-320a-3p
miR-22-3p
miR-26a-5p
other (10)
Ctrl Het KO
KIF2A PPIF IGFBP3 HMGB2 VDAC1
B
norm. mRNA read count
KIF2A VDAC1 PPIF HMGB2 IGFBP3
Ctrl
Het
KO
C
Standardized protein abundance (log2 normalised, centered and scaled)
KIF2A VDAC1 PPIF HMGB2 IGFBP3
Ctrl C4
Het C6
KO C2
D
LIMD1 (protein)
targets (mRNA)
targets (protein)
mRNA or protein expression (z-score)
N T N T N T
E
mRNA or protein expression (z-score)
<0.0001
LIMD1 (protein)
target-set (mRNA)
target-set (protein)
F
mRNA expression (TCGA)
LIMD1-dependent miRNA-target set mRNA (z-score)
LIMD1 mRNA (rpkm)
LUAD
r = -0.47
p = 2.2e-16
N = 517
normal
r = -0.49
p = 9.3e-05
N = 59
G
miRNA target set mRNA z_score Low High
Survival probability
Time (years)
p = 0.0071
H
mRNA expression (TCGA)
LIMD1-dependent miRNA-target set mRNA (z-score)
LIMD1 mRNA (rpkm)
lowest composite score
low composite score
high composite score
highest composite score
I
Group Lowest composite Highest composite
Survival probability
Time (years)
p < 0.0001
J
univariate LUAD-TCGA-mRNA
univariate LUAD-TCGA-mRNA (5-year)
multivariate LUAD-TCGA-mRNA
multivariate LUAD-TCGA-mRNA (5-year)
sub group
gender
age
stage
LIMD1-AGO2:miRNA-target signature
Hazard ratio (HR)
p = 0.691
p = 0.346
p < 0.001
p < 0.001
p = 0.662
p = 0.611
p < 0.001
p < 0.001
p = 0.771
p = 0.109
p < 0.001
p < 0.001
p = 0.828
p = 0.234
p < 0.001
p = 0.001

Figure S8. LIMD1-dependent targets inversely correlate with LIMD1 in normal and LUAD lung tissue and their dysregulation predicts LUAD survival.

(A–C) Chimeric targeting and expression features of the five-gene LIMD1-dependent target-signature set (KIF2A, VDAC1, PPIF, HMGB2, IGFBP3). **(A)** Chimeric read counts across each mRNA 3′UTR for all indicated reproducible AGO2–miRNA interactions, rather than only the selected representative interaction shown in the main figure (Figure 8A). **(B)** RNA-seq mRNA abundance shown as normalised read counts from three CRISPR biological replicates (Ctrl C2/C3/C4), with DESeq2-adjusted P values (***, P<0.001; ****, P<0.0001). **(C)** MS protein abundance shown as log_2-transformed, mean-centred standardised abundance from six independent replicates per genotype using Ctrl C4, Het C6, and KO C2 samples, with FDR-corrected P values (**, FDR<0.01; ****, FDR<0.0001). **(D, E)** LIMD1 protein and target-set mRNA/protein z-scores in paired NAT (N) and tumour (T) samples (n=99) show reduced LIMD1 with increased target expression from NAT to tumour (corrected RM one-way ANOVA); **(D)** heatmap and **(E)** boxplots with median ± IQR and stats. **(F)** Pearson correlation of LIMD1 mRNA with target-set mRNA z-scores in TCGA-LUAD normal and tumour samples. **(G)** Kaplan–Meier 5-year survival in TCGA-LUAD stratified by target-set mRNA signature (high vs low, top vs bottom half); high expression predicts poorer 5-year survival (log-rank *P* values shown). **(H, I)** LIMD1–target set composite score in LUAD tumours: **(H)** scatterplot of LIMD1 mRNA versus target-set expression coloured by composite quartiles (z[LIMD1] – z[target set]); **(I)** Kaplan–Meier survival for highest versus lowest composite score quartiles; low composite score predicts poorer survival (log-rank *P* value shown). **(J)** Forest plots of Cox regression (univariate and multivariate, overall and 5-year) showing tumour stage and high target-set mRNA levels predict poor survival outcomes.

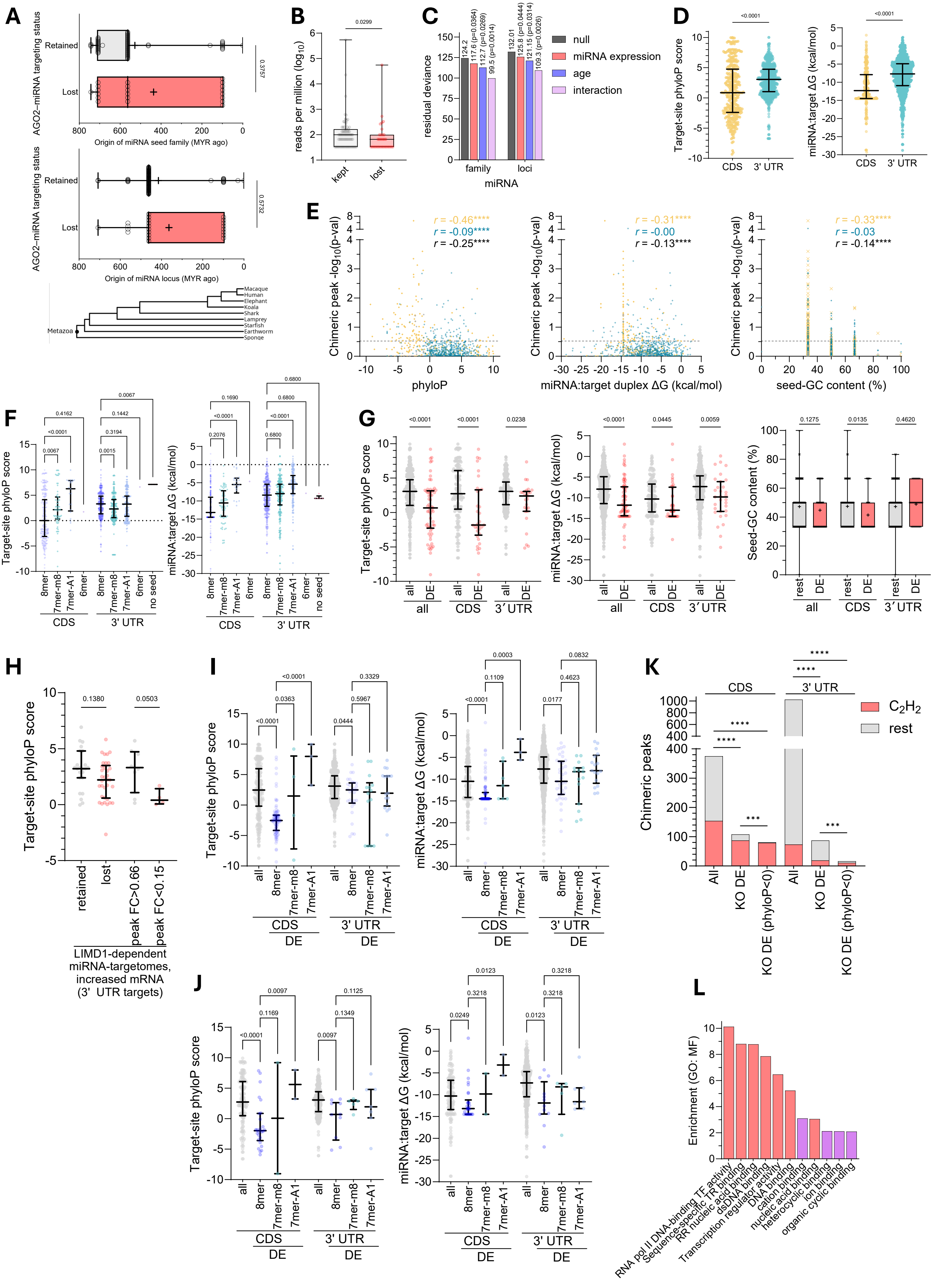

A
AGO2–miRNA targeting status
Retained
Lost
Origin of miRNA seed family (MYR ago)
0.3757
Origin of miRNA locus (MYR ago)
0.5732
Macaque
Human
Elephant
Koala
Shark
Lamprey
Starfish
Earthworm
Sponge
Metazoa
B
reads per million (log10)
0.0299
kept
lost
C
residual deviance
124.2
117.6 (p=0.0364)
112.7 (p=0.0269)
99.5 (p=0.0014)
132.01
125.8 (p=0.0444)
121.15 (p=0.0314)
109.3 (p=0.0026)
family
loci
miRNA
null
miRNA expression
age
interaction
D
Target-site phyloP score
miRNA:target ΔG (kcal/mol)
<0.0001
CDS
3' UTR
E
Chimeric peak -log10(p-val)
r = -0.46****
r = -0.09****
r = -0.25****
phyloP
r = -0.31****
r = -0.00
r = -0.13****
miRNA:target duplex ΔG (kcal/mol)
r = -0.33****
r = -0.03
r = -0.14****
seed-GC content (%)
F
Target-site phyloP score
0.4162
<0.0001
0.0067
0.0067
0.1442
0.3194
0.0015
8mer
7mer-m8
7mer-A1
6mer
no seed
CDS
3' UTR
miRNA:target ΔG (kcal/mol)
0.1690
<0.0001
0.2076
0.6800
0.6800
<0.0001
0.6800
G
Target-site phyloP score
<0.0001
<0.0001
0.0238
all
DE
CDS
3′ UTR
miRNA:target ΔG (kcal/mol)
<0.0001
0.0445
0.0059
Seed-GC content (%)
0.1275
0.0135
0.4620
rest
H
Target-site phyloP score
0.1380
0.0503
retained
lost
peak FC>0.66
peak FC<0.15
LIMD1-dependent miRNA-targetomes, increased mRNA (3′ UTR targets)
I
Target-site phyloP score
<0.0001
0.0363
<0.0001
0.3329
0.5967
0.0444
DE
miRNA:target ΔG (kcal/mol)
0.0003
0.1109
<0.0001
0.0832
0.4623
0.0177
K
Chimeric peaks
CDS
3' UTR
****
***
C2H2
rest
All
KO DE
KO DE (phyloP<0)
J
Target-site phyloP score
0.0097
0.1169
<0.0001
0.1125
0.1349
0.0097
miRNA:target ΔG (kcal/mol)
0.0123
0.3218
0.0249
0.3218
0.3218
0.0123
L
Enrichment (GO: MF)
RNA pol II DNA-binding TF activity
Sequence-specific TR binding
RR nucleic acid binding
dsDNA binding
Transcription regulator activity
DNA binding
cation binding
nucleic acid binding
heterocyclic binding
ion binding
organic cyclic binding

Figure S9. LIMD1 augments evolutionarily young yet thermodynamically strong interactions.

(A) Evolutionary age (MYR) of miRNA seed families and loci categorized as retained or lost based on target engagement in LIMD1 KO versus Ctrl hSAECs. Younger miRNAs trended toward loss, but this did not approach significance (Kruskal–Wallis test). A phylogenetic tree provides context. **(B)** Lost miRNAs trend toward lower expression than retained miRNAs (Mann–Whitney test). **(C)** Logistic regression shows both expression level and evolutionary conservation independently predict miRNA retention. **(D)** Comparison of phyloP scores (>0 = increasing conservation, <0 = accelerated evolution) and free-binding energies between CDS and 3′UTR canonical target sites: CDS sites are less conserved but span a broader phyloP range and form more stable duplexes (more negative energies) (Mann–Whitney test). **(E)** Degree of LIMD1 dependence ($-\log_{10}$ p-value) versus features of canonical interactions. Spearman correlations are shown with phyloP conservation scores at target sites, predicted miRNA:target duplex stability (ΔG, kcal/mol), and 6mer seed-GC content (%). CDS sites (yellow), 3′UTR sites (blue), and other regions (grey; 5′UTR, intron, miRNA, other) are indicated. Yellow, blue, and black r values denote correlations for CDS, 3′UTR, and all sites, respectively; significance indicated by asterisks (****P<0.0001). **(F)** Conservation and binding energies across site types (8mer, 7mer-m8, 7mer-A1) in CDS and 3′UTR regions: CDS 8mers are least conserved, CDS 7mer-A1 most conserved, and 8mers in both regions most stable (Kruskal–Wallis with correction). **(G)** For mRNAs with unchanged or increased abundance ($\log_2$FC>0), the most LIMD1-dependent canonical sites (significantly de-enriched in KO) show lower phyloP scores and more negative free-binding energies than the overall CDS or 3′UTR populations, excluding transcript-level effects (Kruskal–Wallis with correction). **(H)** For LIMD1-dependent miRNAs regulating 3′UTRs (miR-27a-3p, miR-27b-3p, miR-26a-5p, miR-24-3p, miR-181a-5p), sites with greatest chimeric read loss (FC<0.15) in transcripts of unchanged abundance are significantly less conserved than minimally affected sites (FC>0.66) (Kruskal–Wallis with correction). **(I, J)** Among all transcripts **(I)** or those unchanged/increased in abundance **(J)**, the most LIMD1-dependent canonical sites are enriched for poorly conserved, thermodynamically stable 8mers (Kruskal–Wallis with correction). **(K)** LIMD1-dependent sites (de-enriched [DE] in KO) are enriched for C2H2-ZNF genes, particularly in CDS; enrichment is even stronger for sites with phyloP<0 (accelerated evolution) (χ^2 test; ***P<0.001, ****P<0.0001). **(L)** Gene Ontology (Molecular Function) analysis of LIMD1-dependent sites with phyloP<0 shows enrichment for DNA-binding and transcription functions (red) and small molecule/ion binding (purple) (FDR q<0.001; GOrilla, REViGO).

A

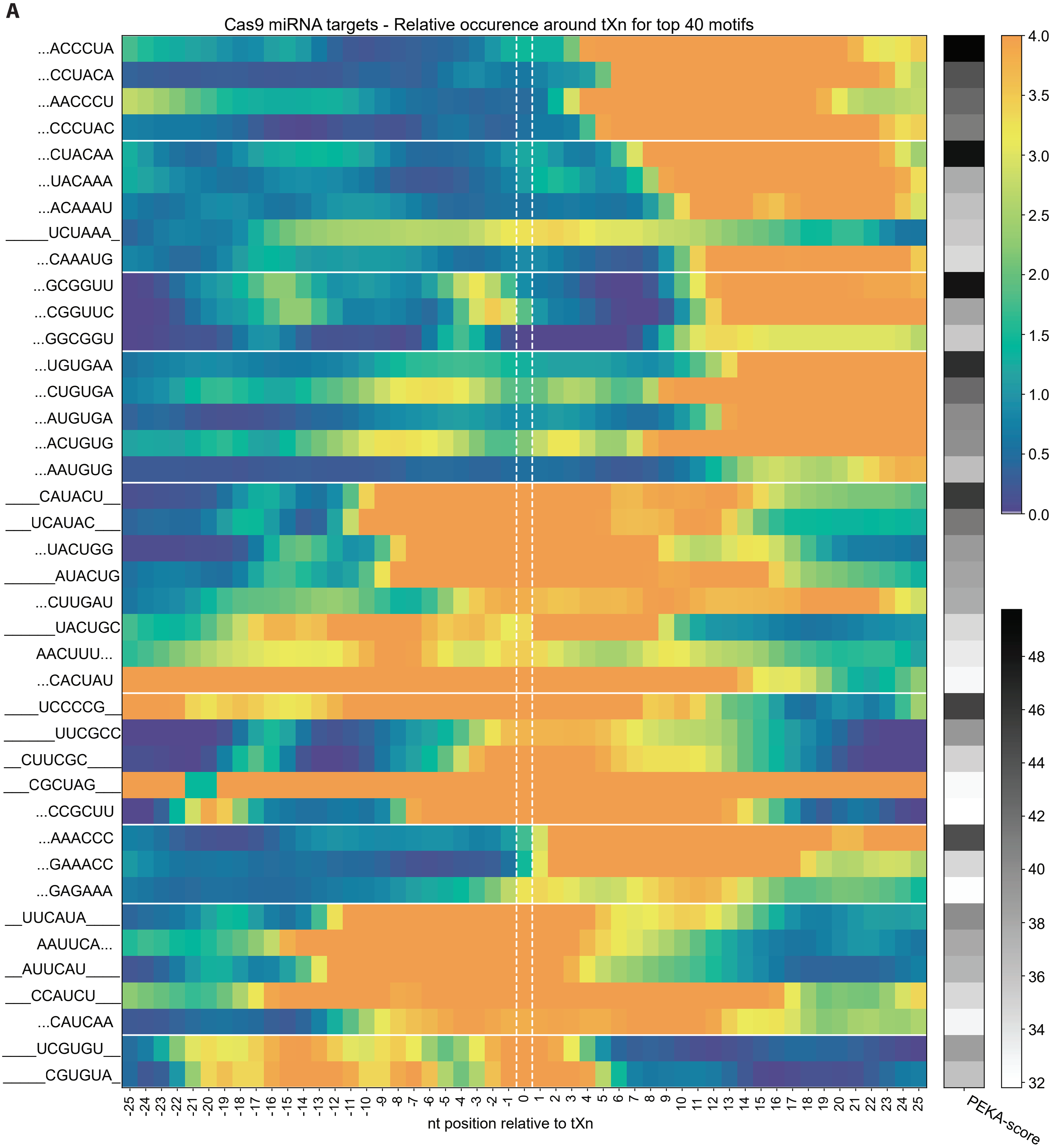

Figure S10. Enlarged views of PEKA heatmaps from Fig. 6A–C

(A–C) Enlarged versions of the (PEKA) heatmaps shown in Fig. 6A–C, corresponding to Ctrl **(A)**, Het **(B)**, and KO **(C).** Heatmaps depict motif relative occurrence (RtXn), defined as fold enrichment of k-mers near thresholded crosslink (tXn) sites relative to distal background. PEKA scores reflect enrichment strength and positional consistency. K-mers are clustered by sequence similarity and aligned by maximal RtXn; dotted lines indicate cases where maximal enrichment occurs >3 nt from the crosslink site. LIMD1 deficiency alters motif identity, positional distribution, and binding footprints.

B

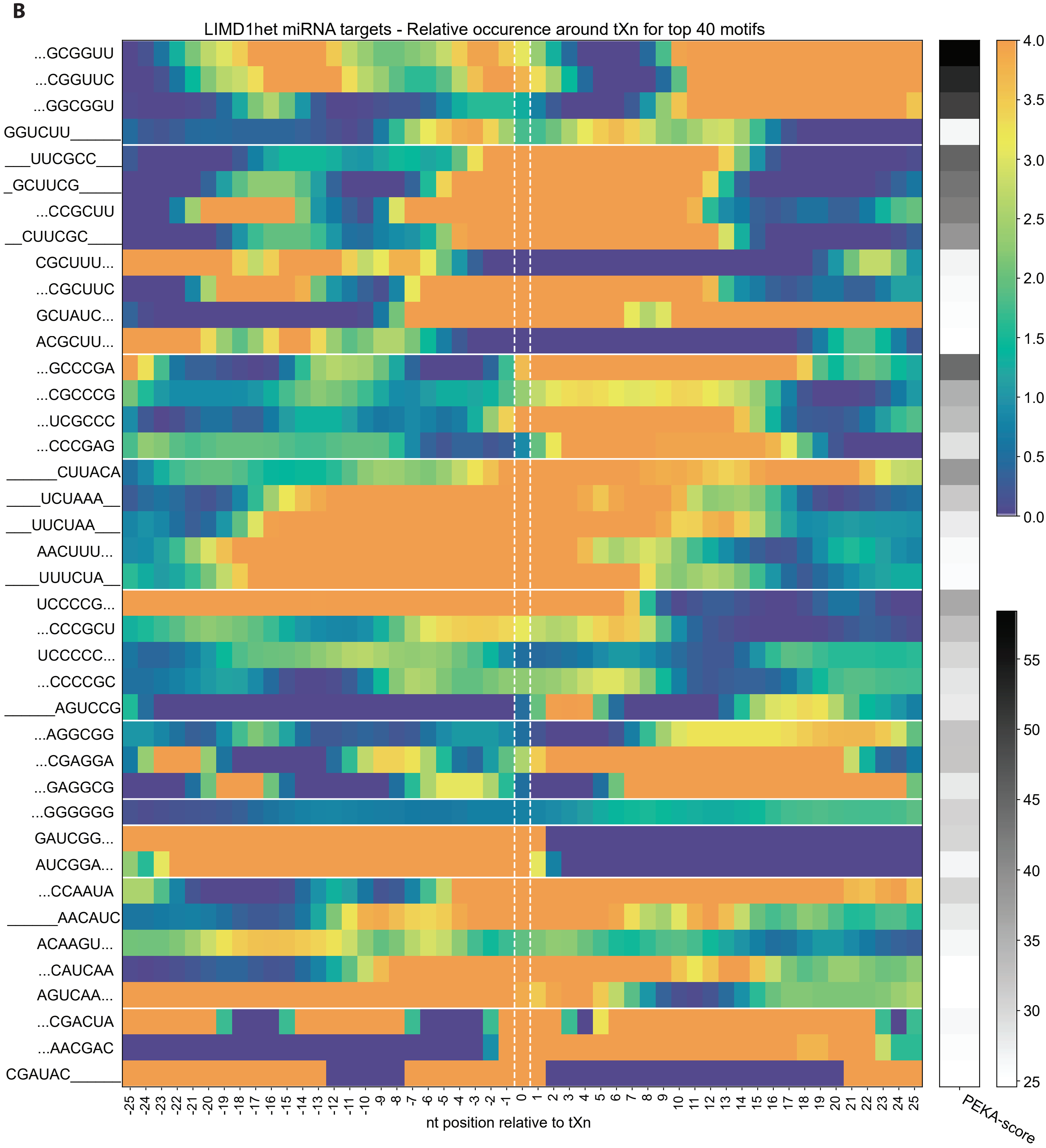
LIMD1het miRNA targets - Relative occurence around tXn for top 40 motifs
...GCGGUU
...CGGUUC
...GGCGGU
GGUCUU______
___UUCGCC___
_GCUUCG_____
...CCGCUU
__CUUCGC____
CGCUUU...
...CGCUUC
GCUAUC...
ACGCUU...
...GCCCGA
...CGCCCG
...UCGCCC
...CCCGAG
______CUUACA
____UCUAAA__
___UUCUAA___
AACUUU...
____UUUCUA__
UCCCCG...
...CCCGCU
UCCCCC...
...CCCCGC
______AGUCCG
...AGGCGG
...CGAGGA
...GAGGCG
...GGGGGG
GAUCGG...
AUCGGA...
...CCAAUA
______AACAUC
ACAAGU...
...CAUCAA
AGUCAA...
...CGACUA
...AACGAC
CGAUAC______
-25 -24 -23 -22 -21 -20 -19 -18 -17 -16 -15 -14 -13 -12 -11 -10 -9 -8 -7 -6 -5 -4 -3 -2 -1 0 1 2 3 4 5 6 7 8 9 10 11 12 13 14 15 16 17 18 19 20 21 22 23 24 25
nt position relative to tXn
4.0
3.5
3.0
2.5
2.0
1.5
1.0
0.5
0.0
55
50
45
40
35
30
25
PEKA-score

C

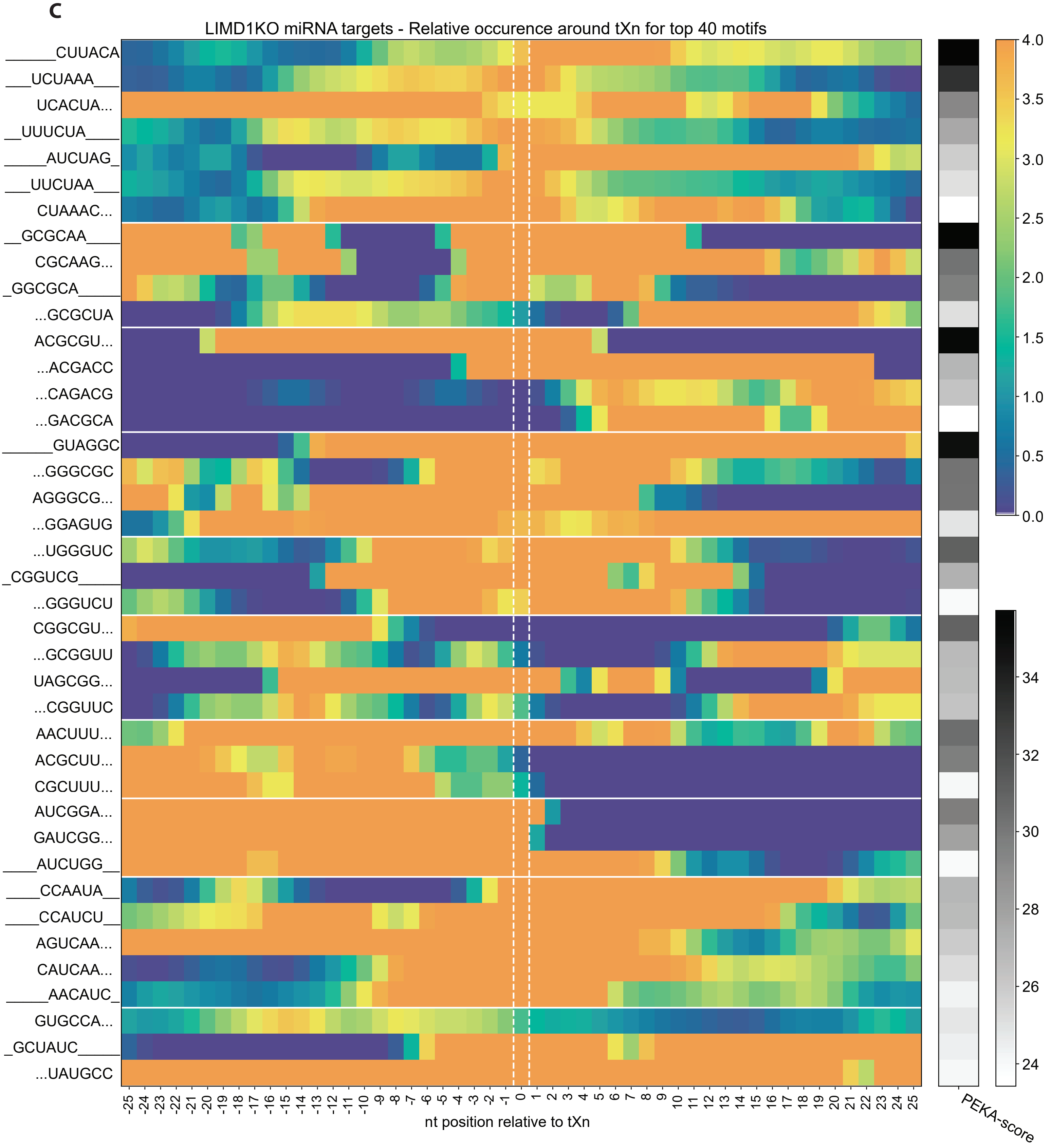

LIMD1KO miRNA targets - Relative occurence around tXn for top 40 motifs
______CUUACA
___UCUAAA___
UCACUA...
__UUUCUA____
_____AUCUAG_
___UUCUAA___
CUAAAC...
__GCGCAA____
CGCAAG...
_GGCGCA_____
...GCGCUA
ACGCGU...
...ACGACC
...CAGACG
...GACGCA
______GUAGGC
...GGGCGC
AGGGCG...
...GGAGUG
...UGGGUC
_CGGUCG_____
...GGGUCU
CGGCGU...
...GCGGUU
UAGCGG...
...CGGUUC
AACUUU...
ACGCUU...
CGCUUU...
AUCGGA...
GAUCGG...
____AUCUGG__
____CCAAUA__
____CCAUCU__
AGUCAA...
CAUCAA...
_____AACAUC_
GUGCCA...
_GCUAUC_____
...UAUGCC
-25 -24 -23 -22 -21 -20 -19 -18 -17 -16 -15 -14 -13 -12 -11 -10 -9 -8 -7 -6 -5 -4 -3 -2 -1 0 1 2 3 4 5 6 7 8 9 10 11 12 13 14 15 16 17 18 19 20 21 22 23 24 25
nt position relative to tXn
4.0
3.5
3.0
2.5
2.0
1.5
1.0
0.5
0.0
34
32
30
28
26
24
PEKA-score

Table S1. Summary of Sanger sequencing identifying editing events of LIMD1-targeted CRISPR-clones.

Sample	Editing event 1	Editing Event 2	Additional info?	Desired Genotype/FrameShift Indicated?
LIMD1 Het C6	1 deletion (T) after gDNA LIMD1 seq base 857	No editing occurred (gDNA LIMD1 seq)	NA	Yes (*LIMD1*$^{+/-}$)
LIMD1 Het C8	5 base deletion (AAACT) at gDNA LIMD1 seq base 854.	No editing occurred (gDNA LIMD1 seq)	NA	Yes (*LIMD1*$^{+/-}$)
LIMD1 Het C11	3 bases before ATG start codon sequence stopped aligning	No editing occurred (gDNA LIMD1 seq)	Mis-alignment indicates large deletion	Yes (*LIMD1*$^{+/-}$)
LIMD1 KO C2	5 base deletion (AAACT) after LIMD1 gDNA base 853	5 base deletion (AAACT) after LIMD1 gDNA base 853	C vs G at bp 900 indicates same editing event on two different alleles	Yes (*LIMD1*$^{-/-}$)
LIMD1 KO C10	1 insertion (T) after gDNA LIMD1 seq base 858	1 base deletion (T) after gDNA LIMD1 seq base 857	NA	Yes (*LIMD1*$^{-/-}$)
LIMD1 KO C20	2 deletions (GC) at gDNA LIMD1 base 859.	2 deletions (CT) at gDNA LIMD1 base 857.	NA	Yes (*LIMD1*$^{-/-}$)

Table S2. Summary of whole genome sequencing (WGS) results probing on-target CRISPR-Cas9-mediated editing events.
Alignment E-values signify number of expected alignments of equivalent or greater score, reflecting strong sequence homology to the guide. VAF = variant allele frequency.

Genotype and clone ID	On-target edit (WGS)	Alignment E-value	WGS evidence	Sanger phasing details
LIMD1 Het C6	1 bp del (CT→C) at Chr 3:45,595,010	4×10^{-5}	30× depth; VAF ~52%	Confirmed
LIMD1 Het C8	1 bp del (CT→C) at Chr 3:45,595,010	4×10^{-5}	30× depth; VAF ~48%	Confirmed
LIMD1 Het C11	4 bp del (TCGCA→T) at Chr 3:45,595,011	2×10^{-5}	33× depth; VAF ~50%	Confirmed
LIMD1 KO C2	5 bp del (GAAACT→G) at Chr 3:45,595,006	9×10^{-6}	32× depth; VAF > 95%	Confirmed
LIMD1 KO C10	Exon-scale deletion (~2 kb)	n/a	≥ 10 split reads & discordant pairs; ~50 % coverage drop	Compound het: +T ins after base 858; –T del after base 857
LIMD1 KO C20	Exon-scale deletion (~2 kb)	n/a	≥ 10 split reads & discordant pairs; ~45% coverage drop	Biallelic 2 bp dels (ΔGC at 859; ΔCT at 857)

Table S3. Summary of Whole genome sequencing results probing putative off-target CRISPR-Cas9 editing events.
Homology E-values signify number of expected alignments of equivalent or greater score, here reflecting weak sequence homology to the guide.

Genotype and clone ID	Putative off-target edit?	Chromosome	Genomic Coordinates (GRCh38, bp)	Deletion length (bp)	Homology E-value	Genomic context
LIMD1 Het C6	Yes	2	Chr 2:62,730,764–62,730,886	122	11	Noncoding
LIMD1 Het C8	No	–	–	–	–	–
LIMD1 Het C11	No	–	–	–	–	–
LIMD1 KO C2	No	–	–	–	–	–
LIMD1 KO C10	Yes	2	Chr 2:62,730,772–62,730,879	107	9.7	Noncoding
LIMD1 KO C20	Yes	2	Chr 2:62,730,764–62,730,853	89	8.6	Noncoding

Table S4.

qc_metrics												
Sample	IP or Input	Initial reads	% pass trim	% rep elements	% uniquely aligned to genome	% PCR duplicates	Final nonchimeric reads	AGO2 clusters	AGO2 peaks	Final chimeric reads	% chimeras	Chimeric clusters
input_WT_R1_001	Input	39,844,858	92.03%	64.80%	33.65%	22.18%	3,379,282					
input_Ctrl_C3_R1_001	Input	65,427,680	99.10%	67.60%	43.77%	18.81%	7,465,573					
input_Ctrl_C4_tr2_R1_001	Input	74,443,442	97.87%	66.91%	40.41%	21.47%	7,650,401					
input_Ctrl_C2_R1_001	Input	39,573,542	87.10%	73.66%	21.62%	21.98%	1,531,132					
input_Ctrl_C4_tr1_R1_001	Input	44,723,159	97.55%	63.75%	34.40%	20.18%	4,343,195					
input_LIMD1_Het_C6_tr1_R1_001	Input	35,619,172	75.60%	64.25%	29.30%	19.65%	2,266,277					
input_LIMD1_Het_C8_R1_001	Input	46,253,733	88.26%	72.28%	23.02%	22.75%	2,011,613					
input_LIMD1_Het_C11_R1_001	Input	72,187,939	94.02%	60.33%	30.83%	23.48%	6,350,342					
input_LIMD1_Het_C6_tr2_R1_001	Input	68,990,119	99.37%	62.19%	36.21%	20.96%	7,418,744					
input_LIMD1_KO_C10_R1_001	Input	43,115,873	90.42%	51.59%	40.98%	20.43%	6,154,821					
input_LIMD1_KO_C2_tr1_R1_001	Input	38,989,398	87.07%	68.23%	27.52%	21.62%	2,325,908					
input_LIMD1_KO_C20_R1_001	Input	66,900,528	98.23%	69.19%	35.90%	20.18%	5,802,502					
input_LIMD1_KO_C2_tr2_R1_001	Input	63,084,068	99.05%	64.02%	43.74%	17.93%	8,070,176					
IP_WT_R1_001	IP	43,185,429	93.18%	35.61%	38.00%	24.82%	7,402,362	203,241	5,127	68,147	0.91%	1,027
IP_Ctrl_C3_R1_001	IP	74,775,141	98.43%	38.13%	28.75%	17.92%	10,745,618	568,912	8,079	87,380	0.81%	1,331
IP_Ctrl_C4_tr2_R1_001	IP	91,933,843	98.72%	36.13%	29.86%	21.59%	13,571,213	568,933	7,409	82,827	0.61%	1,476
IP_Ctrl_C2_R1_001	IP	62,766,609	97.45%	52.35%	27.23%	23.72%	6,052,533	568,924	3,686	67,515	1.10%	1,476
IP_Ctrl_C4_tr1_R1_001	IP	54,872,857	98.01%	40.59%	43.18%	21.71%	10,801,829	568,962	8,729	122,848	1.12%	2,050
IP_LIMD1_Het_C6_tr1_R1_001	IP	58,207,062	96.29%	49.19%	32.77%	22.05%	7,273,949	533,691	4,679	52,724	0.72%	784
IP_LIMD1_Het_C8_R1_001	IP	61,930,112	87.28%	51.63%	27.64%	21.26%	5,691,103	533,699	2,515	29,161	0.51%	526
IP_LIMD1_Het_C11_R1_001	IP	81,083,849	98.28%	46.45%	26.65%	22.15%	8,852,616	533,680	4,347	41,289	0.46%	665
IP_LIMD1_Het_C6_tr2_R1_001	IP	82,135,404	97.84%	38.33%	32.46%	20.85%	12,730,630	533,702	5,395	69,155	0.54%	979
IP_LIMD1_KO_C10_R1_001	IP	45,212,401	92.73%	35.69%	34.53%	21.10%	7,346,652	530,767	4,715	33,595	0.46%	547
IP_LIMD1_KO_C2_tr1_R1_001	IP	47,421,688	93.44%	55.42%	28.17%	20.64%	4,416,552	530,771	3,985	34,583	0.78%	652
IP_LIMD1_KO_C20_R1_001	IP	74,234,778	98.13%	46.76%	26.30%	20.49%	8,111,229	530,808	4,948	51,718	0.63%	758
IP_LIMD1_KO_C2_tr2_R1_001	IP	82,288,388	98.32%	36.42%	28.09%	19.94%	11,567,691	530,794	4,976	51,165	0.44%	703

Table S5.

qc_metrics (summary)												
Sample	IP or Input	Initial reads	% pass trim	% rep elements	% uniquely aligned to genome	% PCR duplicates	Final nonchimeric reads	AGO2 clusters	AGO2 peaks	Final chimeric reads	% chimeras	Chimeric clusters
input_Ctrl	Input	56,041,956	95.41%	67.98%	35.05%	20.61%	5,247,575					
input_LIMD1_Het	Input	55,762,741	89.31%	64.76%	29.84%	21.71%	4,511,744					
input_LIMD1_KO	Input	53,022,467	93.69%	63.26%	37.04%	20.04%	5,588,352					
IP_Ctrl	Input	71,087,113	98.15%	41.80%	32.26%	21.24%	10,292,798	568,933	6,976	90,143	0.91%	1,583
IP_LIMD1_Het	Input	70,839,107	94.92%	46.40%	29.88%	21.58%	8,637,075	533,693	4,234	48,082	0.56%	739
IP_LIMD1_KO	Input	62,289,314	95.66%	43.57%	29.27%	20.54%	7,860,531	530,785	4,656	42,765	0.58%	665

Table S4-5. Quality control metrics of chimeric-eCLIP experiment

4) This table summarizes sequencing and processing statistics for all chimeric-eCLIP libraries (two biological replicates per genotype) and their matched inputs. For each sample, we report:

IP or input: immunoprecipitation (IP) versus input control.
Initial reads: total raw reads obtained.
% pass trim: fraction of reads retained after adapter and quality trimming.
% rep elements: fraction of reads mapping to repetitive elements.
% uniquely aligned to genome: fraction of non-repetitive reads that map uniquely to the reference genome.
% PCR duplicates: fraction of uniquely aligned reads flagged as PCR duplicates.
Final non-chimeric reads: number of unique, non-chimeric reads used for peak calling.
AGO2 clusters: number of enriched binding clusters called genome-wide.
AGO2 peaks: number of reproducible peaks after irreproducible discovery rate (IDR) filtering. A peak is defined as a cluster with log2 fold enrichment > 3 and p-value < 0.001.
Final chimeric reads: number of reads containing chimeric (miRNA–mRNA) junctions.
% chimeras: proportion of final non-chimeric reads that yielded chimeric alignments.
Chimeric clusters: number of unique miRNA–target interaction sites identified.

These metrics confirm high library complexity, efficient removal of artifacts, and robust identification of AGO2–miRNA crosslinked sites across all genotypes, as well as consistency of differences between sample groups. One replicate was performed on WT cells (non-CRISPR-targeted and no single-cell selection) hSAECs for benchmarking.

5) Summary (means) of above per input or IP sample for each sample group.

Supplementary Excel Files

Supplementary Excel file S1

MS_Het and KO v Ctrl (n=6)

Mass spectrometry of proteins in LIMD1Het and LIMD1KO vs Control SAEC

Supplementary Excel file S2

Matched_repro_chimeric peaks_KOvCtrl_phyloP_duplexenergy_analysis

PhyloP scores and duplex binding energies for matched reproducible chimeric peaks in LIMD1KO vs Control SAEC.

Supplementary Excel file S3

mRNA-seq_LIMD1KO_vs_Ctrl_DESeq2_results

Differential expression analysis for mRNA in LIMD1KO vs Control SAEC.

Supplementary Excel file S4

mRNA-seq_LIMD1Het_vs_Ctrl_DESeq2_results

Differential expression analysis for mRNA in LIMD1Het vs Control SAEC.

Supplementary Excel file S5

Reproducible miR-AGO2-chimeric eCLIP peaks_Ctrl_Het_KO

Reproducible chimeric peaks from AGO2 miR-eCLIP in Control LIMD1Het and LIMD1KO SAEC
